# Phage–phage competition and biofilms affect interactions between two virulent bacteriophages and *Pseudomonas aeruginosa*

**DOI:** 10.1093/ismejo/wraf065

**Published:** 2025-04-06

**Authors:** Magdalena Bürkle, Imke H E Korf, Anne Lippegaus, Sebastian Krautwurst, Christine Rohde, Chantal Weissfuss, Geraldine Nouailles, Xavière Menatong Tene, Baptiste Gaborieau, Jean-Marc Ghigo, Jean-Damien Ricard, Andreas C Hocke, Kai Papenfort, Laurent Debarbieux, Martin Witzenrath, Sandra-Maria Wienhold, Gopinath Krishnamoorthy

**Affiliations:** Department of Infectious Diseases, Respiratory Medicine and Critical Care, Charité – Universitätsmedizin Berlin, Corporate Member of Freie Universität Berlin and Humboldt-Universität zu Berlin, 10117 Berlin, Germany; Pharmaceutical Biotechnology, Fraunhofer Institute for Toxicology and Experimental Medicine, 38124 Braunschweig, Germany; General Microbiology, Institute of Microbiology, Friedrich Schiller University, 07745 Jena, Germany; General Microbiology, Institute of Microbiology, Friedrich Schiller University, 07745 Jena, Germany; Clinical Phages and Regulations, Leibniz Institute DSMZ-German Collection of Microorganisms and Cell Cultures, 38124 Braunschweig, Germany; Department of Infectious Diseases, Respiratory Medicine and Critical Care, Charité – Universitätsmedizin Berlin, Corporate Member of Freie Universität Berlin and Humboldt-Universität zu Berlin, 10117 Berlin, Germany; Department of Infectious Diseases, Respiratory Medicine and Critical Care, Charité – Universitätsmedizin Berlin, Corporate Member of Freie Universität Berlin and Humboldt-Universität zu Berlin, 10117 Berlin, Germany; Bacteriophage Bacterium Host, Department of Microbiology, Institut Pasteur, Université Paris-Cité, CNRS UMR6047, 75015 Paris, France; Bacteriophage Bacterium Host, Department of Microbiology, Institut Pasteur, Université Paris-Cité, CNRS UMR6047, 75015 Paris, France; Infection Antimicrobials Modelling Evolution, Université Paris Cité, Inserm, UMR 1137, 75018 Paris, France; DMU ESPRIT, Médecine Intensive Réanimation, APHP, Hôpital Louis Mourier, 92700 Colombes, France; Genetics of Biofilms Laboratory, Department of Microbiology, Institut Pasteur, Université Paris-Cité, UMR CNRS 6047, 75015 Paris, France; Infection Antimicrobials Modelling Evolution, Université Paris Cité, Inserm, UMR 1137, 75018 Paris, France; DMU ESPRIT, Médecine Intensive Réanimation, APHP, Hôpital Louis Mourier, 92700 Colombes, France; Department of Infectious Diseases, Respiratory Medicine and Critical Care, Charité – Universitätsmedizin Berlin, Corporate Member of Freie Universität Berlin and Humboldt-Universität zu Berlin, 10117 Berlin, Germany; General Microbiology, Institute of Microbiology, Friedrich Schiller University, 07745 Jena, Germany; Microverse Cluster, Friedrich Schiller University Jena, 07745 Jena, Germany; Bacteriophage Bacterium Host, Department of Microbiology, Institut Pasteur, Université Paris-Cité, CNRS UMR6047, 75015 Paris, France; Department of Infectious Diseases, Respiratory Medicine and Critical Care, Charité – Universitätsmedizin Berlin, Corporate Member of Freie Universität Berlin and Humboldt-Universität zu Berlin, 10117 Berlin, Germany; German Center for Lung Research (DZL), Berlin, Germany; Department of Infectious Diseases, Respiratory Medicine and Critical Care, Charité – Universitätsmedizin Berlin, Corporate Member of Freie Universität Berlin and Humboldt-Universität zu Berlin, 10117 Berlin, Germany; Department of Infectious Diseases, Respiratory Medicine and Critical Care, Charité – Universitätsmedizin Berlin, Corporate Member of Freie Universität Berlin and Humboldt-Universität zu Berlin, 10117 Berlin, Germany

**Keywords:** bacteria–phage interactions, phage–phage competition, phage therapy, pneumonia, cystic fibrosis, phage resistance

## Abstract

Virulent bacteriophages (or phages) are viruses that specifically infect and lyse a bacterial host. When multiple phages co-infect a bacterial host, the extent of lysis and dynamics of bacteria–phage and phage–phage interactions are expected to vary. The objective of this study is to identify the factors influencing the interaction of two virulent phages with different *Pseudomonas aeruginosa* growth states (planktonic, an infected epithelial cell line, and biofilm) by measuring the bacterial time-kill and individual phage replication kinetics. A single administration of phages effectively reduced *P. aeruginosa* viability in planktonic conditions and infected human lung cell cultures, but phage-resistant variants subsequently emerged. In static biofilms, the phage combination displayed initial inhibition of biofilm dispersal, but sustained control was achieved only by combining phages and the meropenem antibiotic. In contrast, adherent biofilms showed tolerance to phage and/or meropenem, suggesting a spatio-temporal variation in the phage–bacterial interaction. The kinetics of adsorption of each phage to *P. aeruginosa* during single or co-administration were comparable. However, the phage with the shorter lysis time depleted bacterial resources early and selected a specific nucleotide polymorphism that conferred a competitive disadvantage and cross-resistance to the second phage. The extent and strength of this phage–phage competition and genetic loci conferring phage resistance are, however, *P. aeruginosa* genotype-dependent. Nevertheless, adding phages sequentially resulted in their unimpeded replication with no significant increase in bacterial host lysis. These results highlight the interrelatedness of phage–phage competition, phage resistance, and specific bacterial growth state (planktonic/biofilm) in shaping the interplay among *P. aeruginosa* and virulent phages.

## Graphical Abstract

**Graphical Abstract:**
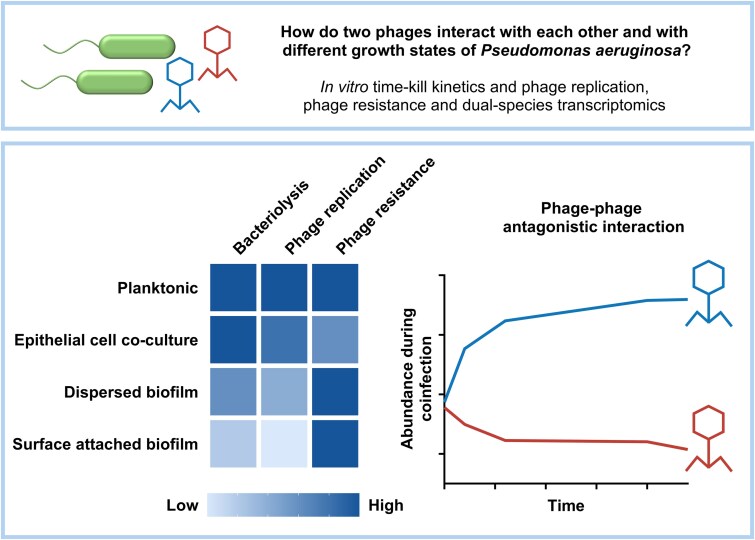

## Introduction

*Pseudomonas aeruginosa* inhabits terrestrial ecosystems but also colonizes and infects humans [1]. The ability of *P. aeruginosa* to form biofilms—aggregates of bacterial cells embedded in an extracellular matrix—allows it to survive under growth-limiting conditions, including hostile immune and antibiotic-induced stress [2, 3]. Naturally occurring biofilms are microcosms of one or more microbial communities, which may include virulent bacteriophages (or phages) [4–6]. From the perspective of addressing the antimicrobial-resistant crisis, the utilization of phages as an adjunct to the antibacterial action of antibiotics and the host response has attracted significant attention [7–9]. Combining multiple virulent phages may be necessary to increase and/or broaden the bacteriolytic activity against diverse clinical isolates and, supposedly, to reduce anti-phage immunity and the emergence of phage resistance [10].

Diffusion-limiting biofilm structures tend to restrict the free interaction between phage(s) and bacteria and/or promote their coexistence [11–16], thereby reducing phage-mediated lysis. Conversely, certain phages encode enzymes capable of degrading the biofilm matrix, cell wall, and nucleic acids or manipulating metabolic functions in *P. aeruginosa* to complete their lytic cycle [17, 18]. Phage infection has been shown to select for mutants with altered phenotypes. For example, a loss-of-function mutation in the *retS* gene—which encodes a histidine kinase that regulates biofilm formation and phage infection—can result in hyper-biofilm formation and phage resistance [19, 20]. Besides biofilm, the other bacterial growth phases and metabolic states, the mammalian immune response, the abundance and genetic composition of phages, and *P. aeruginosa* strain-specific variations in phage resistance mechanisms are among the factors known to determine the outcome of interactions in a given bacteria–phage pair [6, 21].

The capacity of a phage to alter the fitness of co-infecting phages has the potential to modify the collective dynamics of interaction among bacteria and phages, such as lysis-lysogeny switch, competitive exclusion, cross-protection, and temperature-dependent variation in lytic phage traits [22–26]. Understanding the factors that influence such phage–phage interactions and bacterial response to concurrent phage infections is necessary to consider their relevance in natural mixed communities, but also for optimizing phage combinations for therapeutic use.

In this study, we therefore investigated the interaction between *P. aeruginosa* PAO1 planktonic/biofilm growth and two double-stranded (ds) virulent DNA phages, JG005 and JG024 of the genera *Pakpunavirus* and *Pbunavirus*, respectively [27–30]. Based on bacterial killing kinetics and individual phage replication readouts, as well as genome and dual RNA sequencing, our results have identified *P. aeruginosa* genotype, biofilm spatial restriction, phage resistance, and phage–phage competition as interrelated factors influencing interactions between *P. aeruginosa* and two virulent phages.

## Materials and methods

### *P. aeruginosa*–phage interaction

Bacterial culture and phage suspensions were prepared as previously reported [31] (Supplementary Methods). Supplementary Table S1 includes the phage-to-bacteria ratios used for each experiment. A *P. aeruginosa* strain that supports replication of specific or phylogenetically similar lytic phages (as tested in this study) is termed as ‘indicator strain’ and used to enumerate individual phage counts from the combination. Digital images of plates containing plaques (Interscience Scan 4000) were transformed using FIJI ImageJ2 prior to Plaque Size Tool analysis [32]. Bacteriophage-insensitive mutant (BIM) frequencies were determined, as reported earlier [33].

Indicated *P. aeruginosa* strains were cultured in Luria–Bertani broth under shaking conditions and further diluted to OD_600_ ≈ 0.01 (37°C with 5% CO_2_) prior to treatment with either JG005 or JG024 or a combination of both. For sequential treatment, JG024 (1E+07 PFU/mL) was added first, followed by JG005 (1E+07 PFU/ml) 2 h later.

Equal numbers of PAO1 were used for infecting the A549 cell line. Each purified phage preparation was added simultaneously 1 h after infection at 1:10 (low) and 10:1 (high) phage-to-bacteria ratios according to the bacterial number used for the initial infection.

Based on the initial *P. aeruginosa* biofilm viable counts, each phage (JG005, JG024, and Bhz17) at 1:10 (low) and 10:1 (high) phage-to-bacteria ratios was combined and administered. For sequential treatment, JG024 (5E+08 PFU/ml) was added 2 h prior to the addition of JG005 (5E+08 PFU/ml). A measure of 5 μg/ml of meropenem (Sigma-Aldrich) was tested alone or with the phage combination (10:1). Phage buffer-treated samples served as an untreated control. Bacterial and phage enumerations were performed at 6- and 24-h post-treatment for each condition.

### Adsorption assay and one-step growth curve

One-step growth curve and adsorption rate of phage particles were determined as previously described [28, 34]. For the one-step curve, indicated phages and *P. aeruginosa* strains were incubated at a ratio of 1:50 for 10 min prior to sampling, and non-adherent phages were discarded after centrifugation. Virion release at 50 min, since the phage titres plateaued there, was calculated as follows: (phage titre_50 min_ – phage titre_initial_)/(phage titre_initial_). The adsorption constant (K_m_) was calculated from the inverse value of the slope from a linear regression of the natural log-transformed free phages divided by the total amount of infected bacteria [35].

### Phage genome

Genome sequences of JG005 and JG024 have been obtained from GenBank under the accession numbers PP712940.1 and NC_017674.1. JG005 genome was re-annotated using the Centre for Phage Technology Galaxy [36], followed by manual revision with UGENE [37] using default parameters. Candidate tRNA or transfer-mRNA genes were detected using ARAGORN [38] and tRNAscan-SE [39]. The bacterial RNA polymerase was inhibited with rifampicin (400 μg/ml, Sigma-Aldrich) [40].

### Bacteria-phage dual RNA sequencing

Transcriptomic analysis via bulk RNA sequencing was carried out using logarithmic growth of PAO1, which was either infected with phage JG024 alone or with JG024 + JG005 in combination. Samples were collected as reported earlier with some modifications [34].

### Genomic analysis of phage-resistant mutants

Genomic DNA of *P. aeruginosa* phage-resistant and parental PAO1 strains was extracted from overnight cultures and sequenced by Illumina. Polymorphisms were identified with the Snippy programme (https://github.com/tseemann/snippy).

### Statistical analysis

GraphPad Prism 10.4.1 software was used for statistical analysis. The specific statistical tests applied are indicated in the figure legends. *P* values of less than 0.05 were considered statistically significant.

## Results

### Phage treatment results in biphasic killing of *P. aeruginosa* in planktonic culture and in the presence of alveolar epithelial human cells

**Figure 1:**
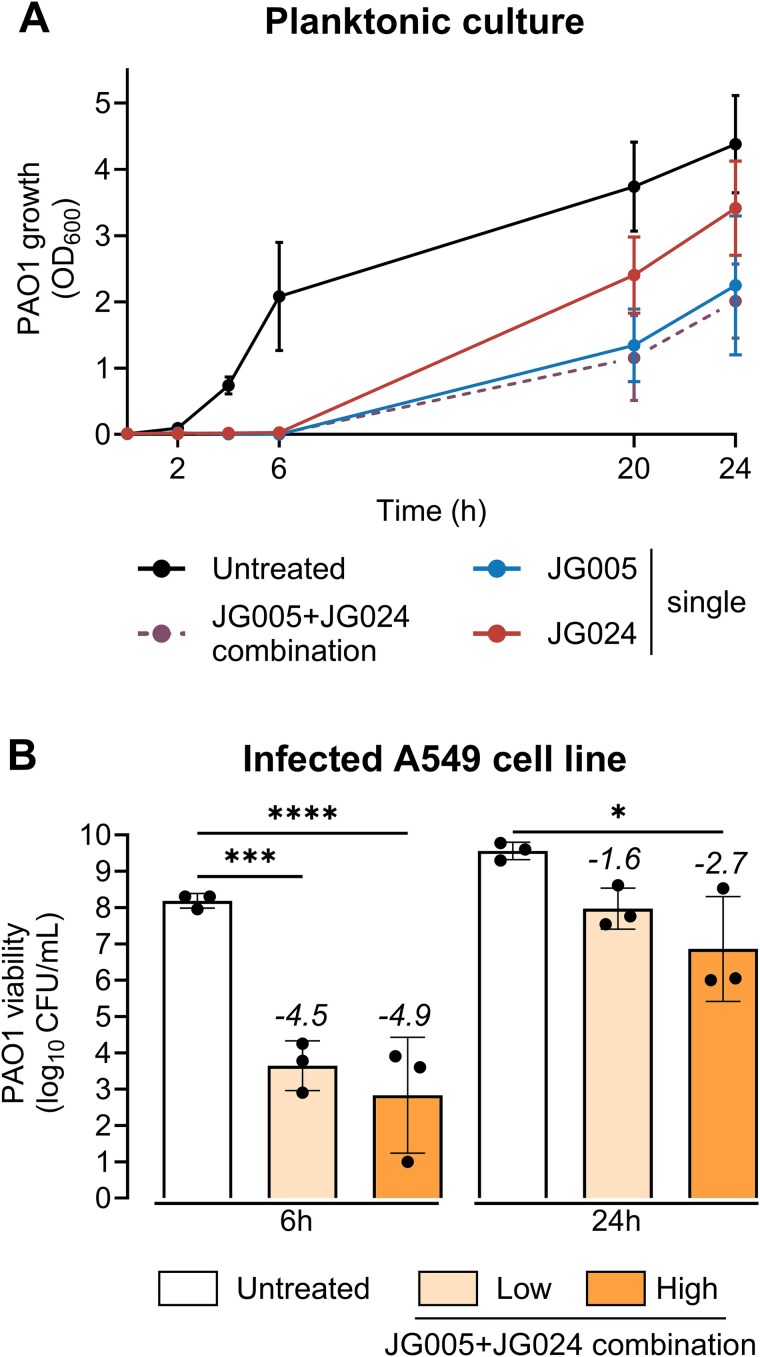
**Time-kill kinetics of phage combinations against different *P. aeruginosa* growth. (A)**  *P. aeruginosa* PAO1 planktonic culture was treated with JG005, JG024, JG005 + JG024 combination (phage-to-bacteria ratio 1:1), or with phage buffer as a control. Bacterial growth was monitored by measuring optical density at 600 nm (OD_600_). **(B)** PAO1 viability from infected A549 human lung epithelial cell line at 6- and 24-h post-treatment with JG005 + JG024 combination and each phage at a high (10:1) and a low (1:10) phage-to-bacteria ratio, or untreated control. Phage treatment was performed 1 h after cell line infection. Numeric values indicate log_10_-fold changes relative to the untreated control. Ordinary one-way ANOVA and Šídák's multiple comparisons test were used to determine *P* values (not significant (*P* > 0.05) is not shown, ^*^P < 0.05, ^***^P < 0.001, ^****^P < 0.0001). The detection limit is 200 CFU/m; all samples below are set to this limit.

JG005 and JG024 phages form clear plaques on the wild-type *P. aeruginosa* PAO1 lawn with mean diameters of 3.94 and 1.22 mm, respectively (Supplementary Fig. S1A). These two phages, when added individually or in combination, effectively lysed the well-stirred planktonic culture of PAO1. The combined lytic effect of JG005 and JG024 was found be similar to that of JG005 (Fig. 1A). All phage treatments showed a biphasic phenotypic spectrum: initial susceptibility followed by growth of a bacterial subpopulation that can evade phage killing at the 24 h time point.

Phages interact with mammalian immune cells, thereby influencing the outcome of bacteria–phage interaction [9, 41]. Therefore, the effect of combining JG005 and JG024 on PAO1 in the presence of the human A549 lung epithelial cell line was assessed (Fig. 1B). Phage treatments resulted in a similar biphasic killing effect in infected A549 monolayers, which was largely comparable to that of planktonic culture conditions. PAO1 growth was reduced at 6 h and then resumed until 24 h. However, the reduced ability of *P. aeruginosa* to evade phage-mediated killing reaffirms the potential role of infected or bystander immune cells in phage–bacteria interactions.

### Combination treatment with JG005 reduced JG024 replication

**Figure 2:**
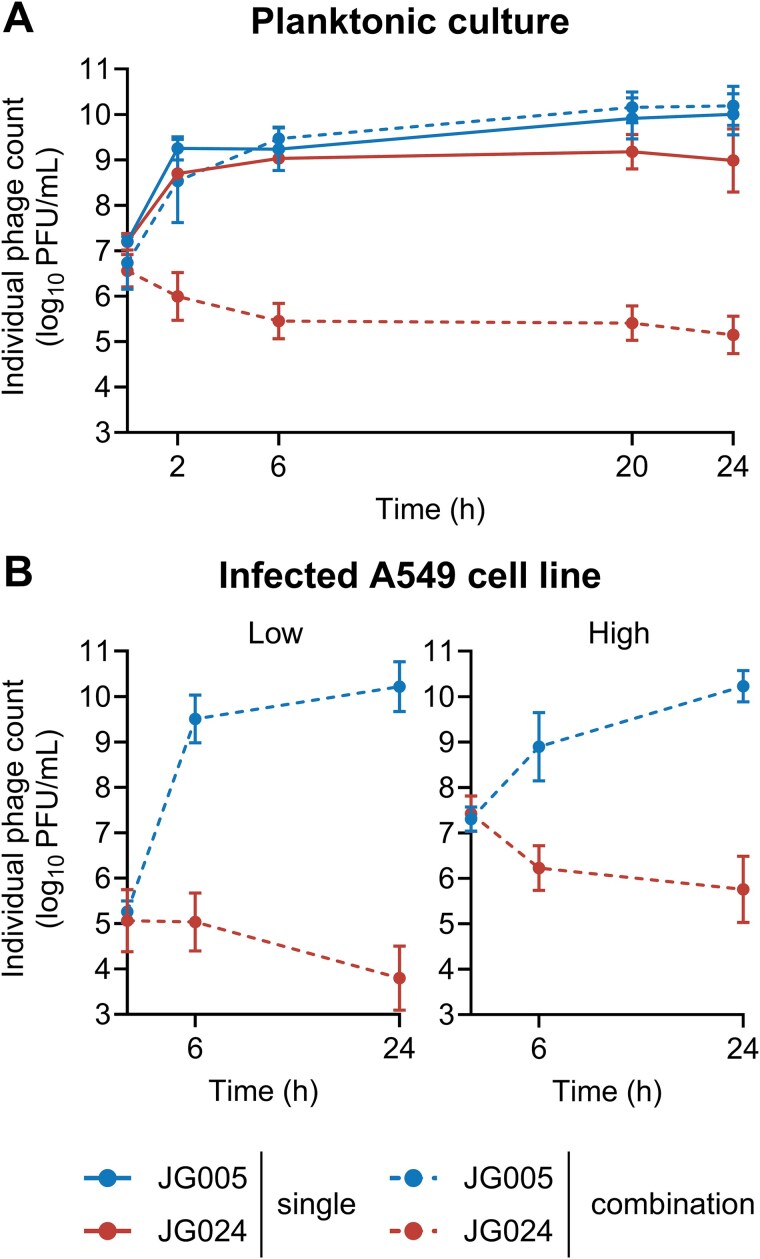
**Replication kinetics of JG005 and JG024 phages. (A)** Both phages JG005 and JG024 were applied individually (single) or in combination at a phage-to-bacteria ratio of 1:1 to *P. aeruginosa* PAO1 planktonic culture or **(B)** in combination with each phage at a low and high phage-to-bacteria ratio of 1:10, and 10:1, respectively, to PAO1-infected A549 human epithelial lung cell line monolayer. Data represent the mean ± SD of *n* = 3 biological replicates. Individual phage titres were assessed by using specific *P. aeruginosa* indicator strains (**listed in**  Supplementary Table S1). Solid lines indicate single-phage treatment, and dotted lines indicate simultaneous combined treatment.

The counts of individual phages from each previous experimental condition were enumerated. JG005 and JG024 numbers showed an increase within two hours when added individually in planktonic culture and then plateaued. At 24 h, JG005 titre (mean 1.36E+10 PFU/ml) was found to be higher relative to JG024 counts (mean 1.89E+09 PFU/ml) (Fig. 2A). In contrast, when combined, only JG005 titre increased, closely matching the titres observed with individual treatment, while the titre of JG024 declined rapidly (mean 1.96E+05 PFU/ml) and then remained in a stalled status. Similar reduction in JG024 replicative fitness was also observed in PAO1 Δ*retS* and clinical *P. aeruginosa* strain CHA [42] planktonic cultures, although JG024 titre in the CHA strain reached the levels observed with single treatment by 24 h (Supplementary Fig. S1B and S1C). A decline in JG024 titre was also observed in the PAO1-infected A549 cell line treated with phage combination (Fig. 2B), indicating a robust negative interaction between JG005 and JG024.

### Latency of JG024 is prolonged in the presence of JG005

**Figure 3:**
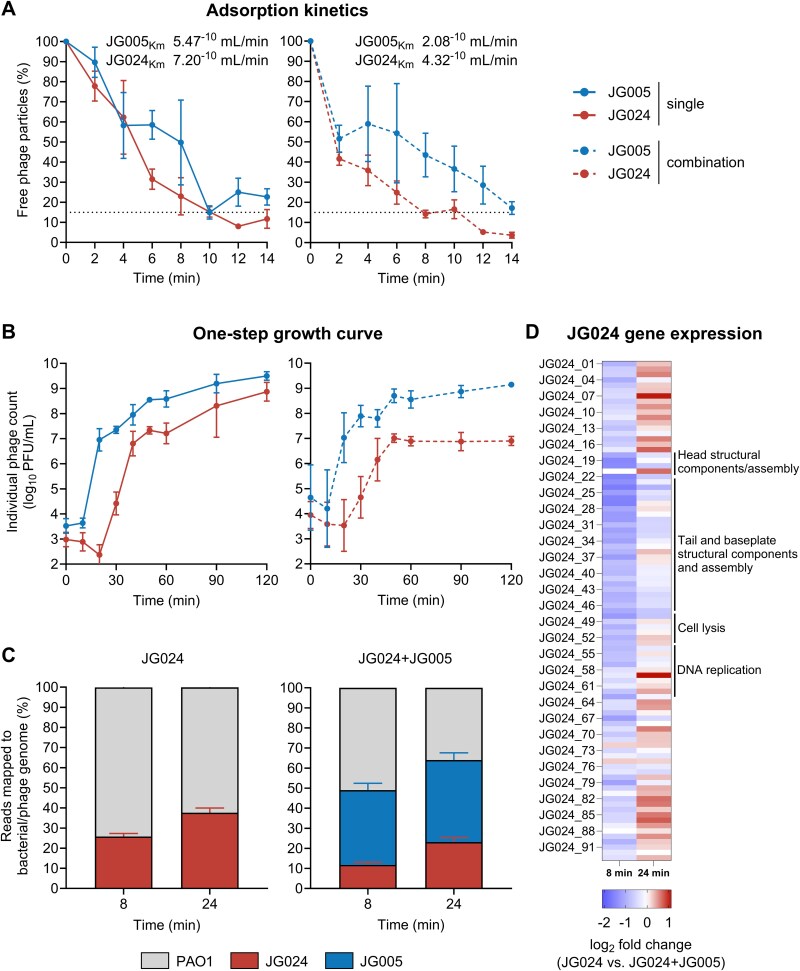
**Growth parameters and transcriptional features of phages when infected with *P. aeruginosa* alone or in combination. (A)** Adsorption kinetics of JG005 and JG024 applied singularly or in combination to *P. aeruginosa* PAO1. The adsorption constant (km) was calculated from the slope of a linear regression of natural-log free phage particles. The horizontal dotted line in the graphs indicates the point at which approximately 85% of phages were bound (in combination: 8 min for JG024, and 14 min for JG005). **(B)** One-step growth curve of phages was applied singularly or in combination. Solid lines indicate single-phage treatment, and dotted lines indicate simultaneous combined treatment. Data of Fig. 3A-B represent the mean ± SD of n = 3 biological replicates. **(C)** Percentage of the total gene reads corresponding to PAO1 and phage gene reads (JG005, JG024) from dual RNA sequencing of PAO1 infected with JG024 or the combination JG024 + JG005 at 8- and 24-min post-infection. RNA sequencing data are presented as mean + SD. **(D)** JG024 + JG005 co-administration data are compared to single infection 8- and 24-min post-infection. RNA sequencing data shown are from two biological replicates.

To understand the negative impact of JG005 on the replicative fitness of JG024, we wondered if competition for access to the likely shared lipopolysaccharide (LPS) receptor could explain the results [27–29]. Contrary to this notion, the adsorption of JG024 was not impaired, but rather better than that of JG005, albeit at a 1:50 phage-to-bacteria ratio, which took an additional 4 min to reach the 85% threshold when co-administered with JG024 (Fig. 3A).

Both phages are presumed to inject their DNA into PAO1 after adsorption, followed by the synthesis of new phage DNA and protein coat, culminating in lysis of the host. By defining these events by one-step growth curves, we calculated that when added individually, 72 and 4 particles of JG005 and JG024, respectively, were released after 50 min of infection (Fig. 3B). On co-administration (input titre of each phage is the same as for single infection), the net increase in JG024 progenies was lower, even though the initial infection rate of both phages increased by 10-fold. Underlying reasons for this observed initial increase in infection rate of both phages remain unclear. A previous study, however, showed that infection by more than one phage can induce the transcription of gene(s) encoding the cognate bacterial receptor(s), which, in turn, correlates with increased adsorption of temperate phages [43]. Nonetheless, the lysis time and number of progenies of JG005 were largely unaffected by infection with JG024. These observations were reproduced with the phylogenetically similar phage pairs, namely JG004 and PTLAW1 (Supplementary Fig. S2A and S2B), suggesting that the relatively shorter latency and increased burst size of JG005 allow it to lyse PAO1 earlier than JG024.

### JG005 depletes host resources prior JG024 maturation and release

Certain viral species are able to prevent the replication of other viral particles that subsequently infect the same host, a phenomenon referred to as ‘superinfection exclusion’ [44]. We tested this possibility by ‘saturatedinfection of PAO1 with high titres of JG024 and subsequently with JG005 2 h later. JG024-infected PAO1 was found to be permissive for subsequent infection and replication of JG005 (Supplementary Fig. S3), although the replication kinetics of both phages were reduced. In contrast, JG005-infected PAO1 significantly decreased the number of released JG024 particles upon successive infection. Of note, the initial drop in JG024 counts suggests its adsorption and DNA injection processes are unperturbed. The presence of any specific JG005-related molecular factor/s disrupting the assembly of JG024 particles is not evident. Therefore, we propose that the faster replication cycle of JG005 might limit the availability of host resources for JG024, which requires a longer latency and maturation period, thereby stalling its replication. Furthermore, it is possible that the JG005-JG024 competition may occur not only in the potentially co-infected PAO1 fraction but also in some of the JG024-only-infected fractions, which may then be re-infected and rapidly lysed by JG005.

### Mutations in an LPS biosynthesis gene confer cross-resistance to JG005 and JG024

*In vitro* assays showed that PAO1 evaded phage killing within 24 h (referred here as BIMs). We reasoned that the nature and frequency of BIMs could explain differences in the killing capacity of the respective phages and phage–phage competition. Therefore, the frequency of BIMs in PAO1 wild-type and PAO1 Δ*retS* was tested in the presence of JG005 and JG024, either alone or in combination. The frequency of JG024-insensitive mutants was 10-fold higher in the Δ*retS* strain than in the PAO1 strain. The BIMs to JG005 or JG005 + JG024 combination treatment arose at a comparable frequency of approximately 1E-07. Notably, the frequency of JG024-insensitive mutants was higher than that of JG005-treated samples for the Δ*retS* strain. The clinical isolate CHA was tested for comparison, and the frequency of mutants was found to be low in response to JG024 or JG024 and JG005 combination (Supplementary Fig. S4A).

After exposure to each phage or the combination, 30 PAO1 BIMs were randomly selected. Of these, only 18 and 11 putative resistant variants of JG005 and JG024, respectively, showed heritable phage resistance. Based on susceptibility profiles, the genomes of 14 representative clones—six JG005-resistant, six JG024-resistant, and two resistant to both phages—were sequenced to identify phage resistance mutations (Supplementary Fig. S4B, Supplementary Table S2).

Four sequenced JG005-resistant PAO1 mutants had mutations in the *wzy* gene, which encodes B and O antigen polymerase required for LPS synthesis*.* The remaining two had missense mutations in *algC* (essential for LPS processing) or *lolC* (encodes lipoprotein localization protein) and were found to be partially resistant to JG005. In contrast, among the six mutants resistant to JG024, three had a missense or frameshift mutation in *wzy,* while the remaining three had a missense or nonsense mutation in *wzz2*, a gene that regulates O-antigen chain length. Finally, both mutants selected against the JG005 + JG024 combination only contained a frameshift mutation in *wzy*. This analysis indicates that a mutation in *wzy* confers cross-resistance to both JG005 and JG024 and that the degree of selective pressure exerted by each infecting phage alone or in combination is different.

To test whether the phage-resistant-conferring mutation is *P. aeruginosa* strain-dependent, we sequenced the genome of three JG024-resistant clones from the PA14 strain background. Notably, these mutants had a missense mutation in the *ssg* gene, which is also involved in LPS synthesis. This observation suggests that the genetic determinants involved in JG024 resistance may differ between *P. aeruginosa* genotypes.

### Transcriptional response suggests early dominance of JG005 over JG024

Specific genomic traits of a phage can influence its competitive interaction. Indeed, our analysis showed that JG005 encodes for at least 90 more genes with at least 15 tRNAs than tRNA-deficient JG024, which may reduce JG005's host dependency with shorter latency (Supplementary Fig. S5A and S5B). Although JG005 encodes additional genetic components, its replication, like that of JG024, is impaired in RNA polymerase-inhibited PAO1 (rifampicin-treated). No such defects were observed in the solvent control, implying that each phage requires bacterial transcriptional components (Supplementary Fig. S5C).

Dual RNA sequencing was performed to find any potential mechanism by which JG024 assembly is specifically disrupted by comparing the transcript abundances between uninfected, JG024-infected, and JG005 + JG024-infected PAO1 planktonic culture (Supplementary Table S3).

PAO1 transcripts were reduced more under JG024 + JG005 co-administration conditions, either due to the combined lytic effect of two phages or predominantly by JG005 (Fig. 3C). Compared to single infection data, the number of JG024 reads dropped by about 54% and 38% at 8 and 24 min, respectively, when co-administered with JG005. The observed drop in JG024 and PAO1 mRNA levels appears to demonstrate a robust correlation with phenotypic data, indicating that JG005 manifests accelerated replication kinetics, suppressing JG024 replication with increased bacteriolysis. When compared with the JG024-alone data, the expression of JG024 genes in JG024 + JG005 infected samples was reduced at 8 min. In contrast, the expression levels of the majority of JG024 genes were found to be comparable between single- and two-phage-infected samples at 24 min, suggesting a transcriptional recovery (Fig. 3D). When this is correlated with the consistent decline observed for JG024 counts in other experiments (e.g. Fig. 2A and Supplementary Fig. S3), it indicates that the JG005-mediated bacterial lysis or net increase in JG005 and JG024 numbers due to co-administration depleted the resources necessary for subsequent steps involving the packaging and release of JG024 virions.

A total of 17 genes were found to be upregulated in all phage-infected samples compared to the uninfected control at both time points. These include *lecA*, phage shock proteins (*PA3728-PA3732*), and cyclic diguanylate-regulated protein secretion (*PA4623-PA4624*), implying changes in the bacterial outer membrane including LPS [45–47]. Upregulation of bacterioferritin B and glutaryl-CoA dehydrogenase genes perhaps indicates an underlying metabolic change to phage infection [48].

Among the six downregulated genes were those of the alkaline protease secretion system (*aprE* and *aprI*), a predicted lipid carrier protein. Furthermore, at 24 min post JG024 or JG024 + JG005 infection, genes associated with energy metabolism, hydrogen cyanide synthesis, and several nutrient transport systems were found to be downregulated, suggesting the onset of phage-mediated metabolic downshift and cell wall modification associated with cell lysis and/or transcriptional regulation promoting evasion of phage attack. Given that several of these genes were also found to be differentially regulated in *P. aeruginosa* infected with phage PAK_P4 (closely related to JG005), ɸKZ, PA5oct, and LUZ19 [34, 49–51], these transcriptional signatures may represent a general response of *P. aeruginosa* to dsDNA phage infection.

A unique subset of 149 genes was found to be differentially expressed only in JG024-infected PAO1 at 8- or 24-min post-infection. Among these, an increased expression of the potassium transporters (*kdpA, kdpF, kdpK*), pyoverdine-iron importers (*PA4364-PA4365*), ribonucleotide reductases, fimbriae synthesis, and a decreased expression of outer membrane proteins porin (*oprB*) and of some heme biosynthesis genes. Most of these JG024-specific transcriptional signatures were absent in JG024 + JG005 co-administered samples. The negative regulators of alginate synthesis, *wzz* (O-antigen chain length regulator), and *PA0909* (initiates autolysis) were among the genes increasingly expressed, consistent with other phage-infected *P. aeruginosa* strains [34]. The expression of genes involved in nutrient transport and in signalling pathways (e.g. c-di-GMP phosphodiesterase) was found to be decreased at both time points. The transcriptional profile of the JG024 + JG005 co-administration induced expression of a distinct set of membrane and metabolic stress-associated genes. Accompanied by the notable absence of many JG024 single infection-specific gene signatures, this observation suggests that JG005 predominantly drives the transcriptional reprogramming during co-administration with JG024.

### Phage–*P. aeruginosa* interaction altered by biofilm spatio-temporal constraints

**Figure 4:**
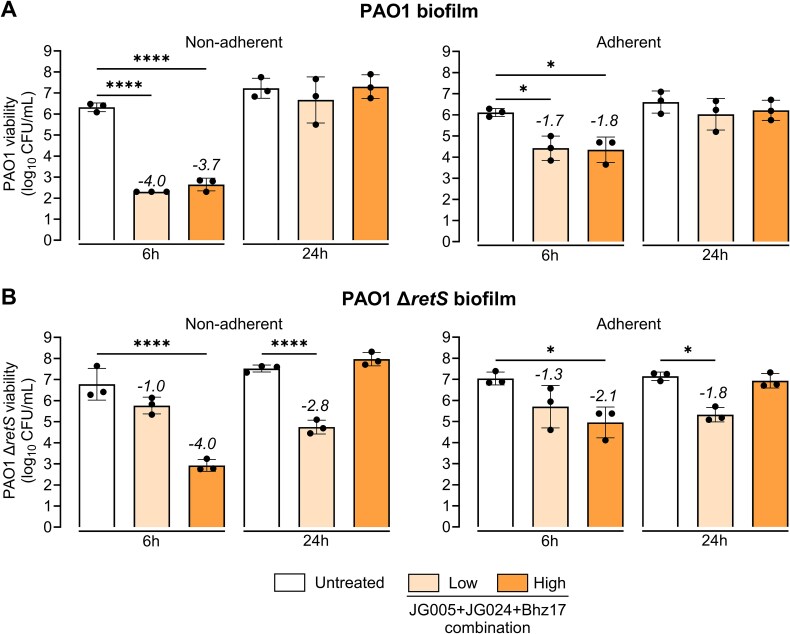
**Time-kill kinetics of phage combination against *P. aeruginosa* biofilms.** Viability of PAO1 from **(A)** non-adherent (dispersed) and adherent (surface-attached) biofilm phases, at 6- and 24-h post-treatment with 3-phage (JG005 + JG024 + Bhz17) combination. Bhz17 is a phage that cannot replicate or lyse PAO1 or its mutant derivative. Viability of **(B)** PAO1 Δ*retS* from non-adherent and adherent biofilm phases 6- and 24-h post-treatment with 3-phage combination. Data represent the mean ± SD of *n* = 3 biological replicates with technical duplicates. Numeric values at the top of the columns indicate log_10_-fold changes relative to the untreated control. Ordinary one-way ANOVA and Šídák's multiple comparisons test were used to determine *P* values (not significant (*P* > 0.05) is not shown, ^*^P < 0.05, ^***^P < 0.001, ^****^P < 0.0001). The detection limit is 200 CFU/mL; all samples below are set to this limit.

To test the hypothesis that bacterial susceptibility, phage replication kinetics, and associated phage–phage competition vary in biofilms, we performed time-kill kinetics and monitored each phage replication on a 24-h-grown PAO1 biofilm in microtitre plates (Supplementary Fig. S6A). Unlike prior tested conditions, Bhz17 was included in the JG005 + JG024 combination for biofilm experiments. Bhz17 is a dsDNA phage—that cannot infect, replicate, and lyse the PAO1 strain (Supplementary Fig. S1A, Supplementary Fig. S6B)—and thus served as a control to determine its ability to compete with JG005 and JG024 or modify their interaction kinetics. This 3-phage combination also mimics some phage therapy practices of using pre-designed, multi-phage cocktails that may include one or more non-replicating phages. Bacterial and individual phage counts were determined from two separate biofilm phases: adherent (surface-attached) and non-adherent (dispersed), to determine spatio-temporal differences, if any. After reduction of the viable PAO1 counts at 6 h, a subpopulation grew to the level of the untreated control after 24 h in both the adherent and the non-adherent phases, regardless of the initial phage concentration used (Fig. 4).

Viable PAO1 counts in the non-adherent phase were lower compared to the adherent phase at 6 h after 3-phage combination treatment (Fig. 4A), indicating effective prevention of biofilm dispersion by phage treatment and insignificant effect on the adherent population. No impact of the phage-to-bacteria ratio was noted in terms of bacterial viability reduction at this time point. These results reveal a spatio-temporal pattern in phage-mediated bacteriolysis, namely, a minimal impact on adherent population viability but an early inhibition of the biofilm dispersal.

To assess the effect of the 3-phage combination on biofilms with increased thickness, a hyper-biofilm-producing *P. aeruginosa* PAO1 Δ*retS* strain [52] was used. Biomass production, quantified by crystal violet staining, was found to be 2.4-fold higher in the untreated Δ*retS* biofilm compared to the PAO1 strain (Supplementary Fig. S6C). Bacterial counts in the Δ*retS* biofilm were increased correspondingly (Fig. 4B). The viability reduction kinetics of Δ*retS* biofilm upon 3-phage combination treatment (each phage at a high phage-to-bacteria ratio) were comparable to those of the PAO1 strain at 6- and 24-h post-treatment. Counterintuitively, treatment with a low phage-to-bacteria ratio reduced the Δ*retS* adherent and dispersed biofilm population over 24 h, although the initial effect at 6 h was lower than in the PAO1 strain. Hence, the observed differences in phage lytic activity against the biofilm of the *retS*-deficient strain can be attributed to either a different phage stoichiometry, delayed killing and/or the growth of phage-resistant clones, or to the RetS regulatory and phage resistance mechanism itself [53].

### Phage abundance varies with biofilm phase

**Figure 5:**
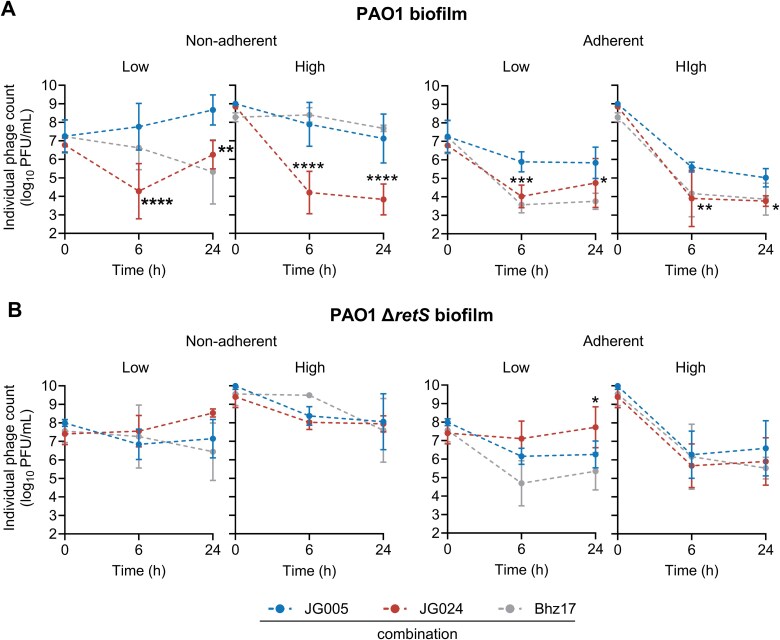
**Replication kinetics of JG005, JG024, and Bhz17 in biofilms. (A)** Phage titres in *P. aeruginosa* PAO1 biofilms and **(B)** PAO1 Δ*retS* biofilms are shown. In the left panels, the number of individual phages of the non-adherent compartments is plotted. Data related to adherent biofilm compartments are shown in the right panels. Data represent the mean ± SD of *n* = 3 biological replicates with technical duplicates. Individual phage titres were assessed by using specific *P. aeruginosa* indicator strains (**listed in**  Supplementary Table S1). Statistical significance was assessed using two-way ANOVA and Dunnett's multiple comparison test to determine *P*-values, comparing 6- and 24-h time points (not significant *P* > 0.05) is not shown, ^*^P ≤ 0.05, ^**^P ≤ 0.01, ^***^P ≤ 0.001, ^****^P < 0.0001). Note that Bhz17 is unable to replicate in the PAO1 strain and is therefore serving as a non-replicative control.

Phage titres in the separate phases of a PAO1 biofilm after combined treatment were also subsequently examined. JG005 titres increased slightly in the non-adherent biofilm phase (Fig. 5A) at the low phage concentration. JG024 titres rapidly decreased by approximately two orders of magnitude, particularly at the high phage concentration, similarly to what we observed in planktonic conditions, and were occasionally even lower than titres of the PAO1-non-replicative Bhz17. The phage titres in the adherent biofilm showed a similar pattern but dropped even lower than in the non-adherent phase.

No defect in JG024 fitness was detected after treatment of Δ*retS* biofilm phases with 3-phage combination: at the high phage-to-bacteria ratio, JG024 abundance was comparable to that of JG005, while at the low ratio, JG024 numbers were even found to have increased (Fig. 5B). This may explain the more sustained killing at low phage concentrations, particularly in the non-adherent phase (Fig. 4B). Together, these data show that JG005 and JG024 replication and interaction also vary within biofilm phases and are influenced by bacterial gene function and phage-to-bacteria ratio.

### Sequential administration prevents JG005-JG024 competition

**Figure 6:**
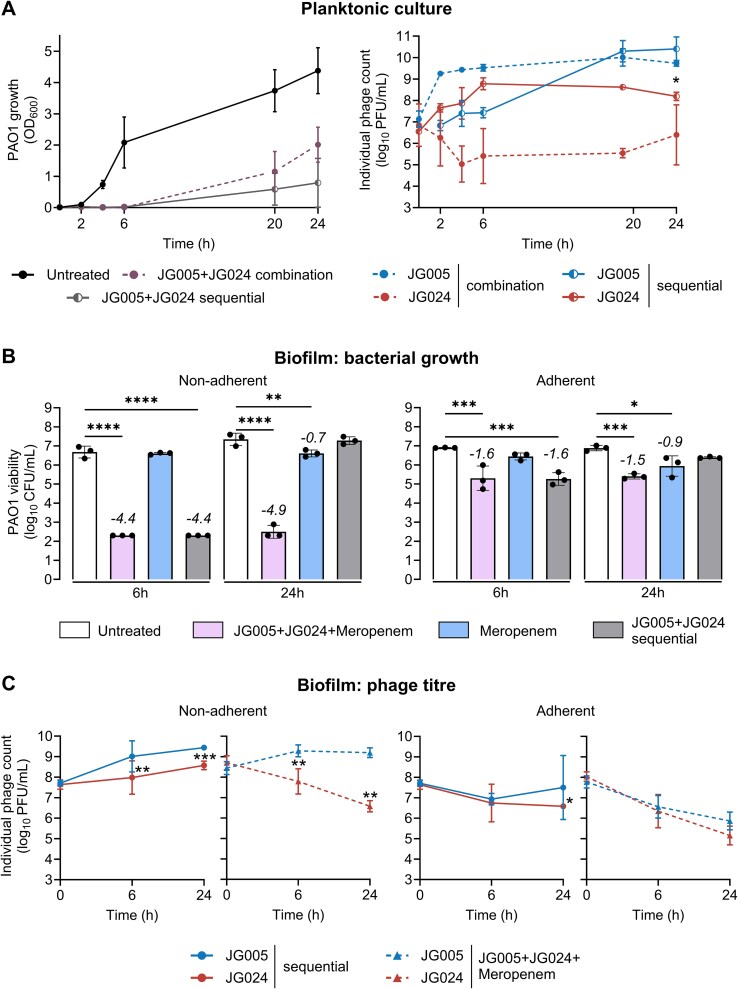
**Effects of sequential phage administration and phage–antibiotic combinations against *P. aeruginosa*.** For sequential and simultaneous phage administration in planktonic culture, both phages were added either simultaneously or JG024 was applied sequentially 2 h before JG005. **(A)** Bacterial growth in response to simultaneous or sequential phage treatment of *P. aeruginosa* PAO1 planktonic culture. Phage replication patterns of JG024 and JG005 are shown. Simultaneous treatment is shown in dotted lines, and sequential phage administration is shown in solid lines. Statistical significance was determined using one-way ANOVA and Šídák's multiple comparisons tests, comparing simultaneous and sequential treatment conditions at 24 h time point, ^*^P < 0.05. Bacterial and phage counts of PAO1 biofilms were enumerated 6 and 24 h with either phages added sequentially, or with a combination of phage+meropenem. **(B)** Bacterial viability of non-adherent population and adherent compartment of static PAO1 biofilm of sequential phage-treated (first JG024, two hours later JG005), phages and meropenem (5 μg/mL) combination, and meropenem alone (5 μg/mL). **(C)** The corresponding phage titres of both non-adherent and adherent compartments are plotted. Sequentially administered phages are shown in solid lines (JG005 and JG024), and a simultaneous combination with meropenem is shown in dotted lines and triangles. Data represent the mean ± SD of *n* = 3. Numeric values indicate log_10_-fold changes relative to the untreated control. Statistical significance was assessed using ordinary one-way ANOVA to determine *P*-values for bacterial viability and two-way ANOVA and Šídák's multiple comparisons test comparing 6 and 24-h time points for phage viability (not significant (*P* > 0.05) is not shown, ^*^P < 0.05, ^**^P < 0.01, ^***^P < 0.001, ^****^P < 0.0001). Detection limit is 200 CFU/mL; all samples below are set to this limit.

In planktonic conditions over 24 h, when JG024 was added 2 h before JG005, bacterial growth tended to be slightly more inhibited without affecting JG024 replication (Fig. 6A). In both biofilm phases, the sequential phage treatment led to the same outcome (Fig. 6B) as the combination treatment (Fig. 4A). Nevertheless, the addition of JG024 prior to JG005 was proven effective in circumventing the competition between phages for available host resources (Fig. 6C).

### Phages and meropenem combination restrict biofilm dispersion

The combination of phage and antibiotics has been shown to increase the efficacy of bacterial killing [7, 8, 18, 54]. To test the impact of high concentrations of meropenem (frequently used for treating severe infections in critically ill patients) on phage-mediated killing and JG005-JG024 competition in biofilm, we included two treatment groups: meropenem alone and a phage+meropenem combination. No reduction of bacterial growth was observed in the meropenem-only group. However, when meropenem and phage were added together, PAO1 growth was restricted over 24 h in the non-adherent phase. A minimal reduction of bacterial viability at 24 h was observed in the adherent phase, suggesting that the combination of phage+meropenem was proven effective in preventing biofilm dispersal (Fig. 6B). The addition of meropenem, however, did not alleviate JG024-JG005 competition, particularly in the non-adherent phase where JG024 was found to be reduced (Fig. 6C).

## Discussion

Our data show that the interactions between two virulent phages vary according to the different *P. aeruginosa* growth phases. In the context of biofilms, adherent (surface-attached) cells exhibited tolerance, while the non-adherent (dispersed biofilm) bacteria were initially relatively susceptible, but subsequently, a population evading phage killing emerged. Such spatio-temporal variations in phage effects and differences in phage resistance evolution can be observed not only in biofilms but also in soil, the plant rhizosphere, and the gut lumen [55–57]. In addition to acting as physical barriers [11–15], additional biofilm-specific factors that might contribute to the recalcitrance of *P. aeruginosa* PAO1 to phage lysis have been previously described: a subpopulation of bacteria in biofilms tends to lose B-band and O-antigen of LPS, which is the putative receptor for JG005 and JG024. Compared to planktonic growth, slow-growing populations in biofilm may undergo specific metabolic changes, including cyclic adenosine monophosphate production, that have been shown to reduce bacterial susceptibility to phage infection and lysis [58, 59]. Finally, transcriptional counter-responses or mutation acquisition under phage-induced stress can influence a subset of genes or select hypermorphic mutations that increase biofilm formation, thereby overturning the initial phage-mediated anti-biofilm effects [60, 61].

The combination of meropenem and JG005 + JG024 treatment strongly inhibited the dispersal of resistant/tolerant biofilm populations to both agents. The relatively low or delayed emergence of bacterial outgrowth that can withstand phage killing in infected A549 cells reinforces the role of immune cells in augmenting phage killing with the reduced ability of phage-resistant variants to survive in an immune milieu [9, 41, 55]. These *in vitro* findings are consistent with some clinical case reports showing improved treatment efficacy when phage combinations are used to synergize antibiotics and immune functions in difficult-to-treat patient groups [62].

Although the titres of JG005 and JG024 were matched prior to simultaneous treatment in our experiments, their ability to co-infect, assemble structures, and complete their lytic cycle was found to be asymmetric. As a result, replication of the ‘weaker’ phage was stalled, especially at a high phage-to-bacteria ratio, due to the depletion of host resources. This observation is reminiscent of ‘interspecific competition’, a phenomenon that occurs across different species, including phages and eukaryotic viruses [5, 23, 24, 63, 64]. While there are different contexts and conditions that can lead to competition, intrinsic features of the phage itself are a major determinant [65–67]. For example, dsDNA phages, with a short latency period and a high burst size, encoding their own set of tRNAs may reduce the dependence of phages on the bacterial host to synthesize their macromolecules and allow evasion of tRNA-targeting host defences. The JG005 genome fulfils these criteria, likely contributing to its superior replicative fitness and its respective dominance over JG024. The resulting asymmetric selection pressure exerted by each phage appeared to alter the spectrum of phage resistance mutations in our study, as reported previously for clinically approved drugs [68]. At early time points in our experiments, the selective pressure on PAO1 was likely to have been exerted predominantly by JG005, leading to resource monopolization and strong selection for JG005 mutants, which also showed cross-resistance to JG024, leading to sustained attenuation of JG024 replication.

Phage–bacteria interaction is *P. aeruginosa* strain-specific; this is reaffirmed by the observations made in *P. aeruginosa* strain CHA, where the initial impairment in JG024 replication was subsequently restored, in contrast to PAO1 strain. We have shown that the frequency of BIMs was strikingly lower in the clinical CHA strain than in PAO1 strain. This may have reduced the conflicts in the replication of JG005 and JG024 and/or with the different underlying mechanisms of phage resistance in the CHA strain and in the Δ*retS* PAO1 [20] strain in planktonic and/or biofilm conditions. Reinforcing this view, our analysis showed that gene mutation conferring JG024 phage resistance is not identical in *P. aeruginosa* strains PAO1 and PA14. Such strain-specific alterations in the mutational spectrum are not uncommon [69]. Thus, *P. aeruginosa* genotype (and specific anti-phage defence systems) may influence the strength of selective pressure exerted by individual phages, thereby altering dynamics of bacterial evolutionary adaptation to phages and phage–phage competition.

Sequential administration of JG024 and JG005 eliminated their competition but may also have influenced the development of phage cross-resistance in our experimental conditions. A previous study [70] showed that only sequential addition of two LPS-binding phages resulted in the acquisition of several resistance mutations in PAO1. Another independent study found that the simultaneous application of four phages was either equally effective or even superior to sequential application in reducing PAO1 viability and mitigating phage resistance [71]. Such sequential or alternating doses of antibiotics have been found to be a promising strategy to increase bacterial susceptibility and prevent the emergence of resistance [72–74]. As we used different phages and bacterial strains, direct comparison of our results with others is not appropriate. However, optimizing the dose, frequency, and order of administration of a compatible phage combination might be critical for sustained bacterial killing with reduced cross-resistance and phage–phage competition.

Inherent limitations of our experiments are the short observation period and the inability to evaluate other confounders, such as coexistence with polymicrobial communities, immune response, route, and schedule of phage administration. Our results thus probably overestimate the frequency and significance of phage resistance and phage–phage competition compared to the context of human disease and terrestrial conditions. For example, in laboratory conditions, phage-resistant mutants deficient in the production of the key virulence factor LPS evolve rapidly. Should this phenomenon occur *in vivo*, it could potentially diminish the capacity of bacteria to survive longer in higher organisms [62, 75]. Furthermore, this study did not examine the impact of the non-replicative Bhz17 phage on phage competition and bacterial adaptive behaviour.

In conclusion, the study results illustrate the influence of altered bacterial growth state and/or individual phage replication traits on the dynamics of bacteria–phage interaction. Future investigations should include increased complexity recapitulating more natural and/or clinically relevant conditions to gain mechanistic insights into multi-species interactions and to develop better health interventions (e.g. phage-mediated pathogen clearance or microbiota manipulation).

## Supplementary Material

Figure_S1_wraf065

Figure_S2_wraf065

Figure_S3_wraf065

Figure_S4_wraf065

Figure_S5_wraf065

Figure_S6_wraf065

Supplementary_Table_S1_wraf065

Supplementary_Table_S2_wraf065

Supplementary_Table_S3_wraf065

Raw_data_set_wraf065

Supplementary_Methods_wraf065

## Data Availability

RNA and DNA sequencing data are available from the NCBI GEO database (GSE271537) and Open Science Framework repository service (doi:10.17605/OSF.IO/RZKDH), respectively. All other data generated or analysed during this study are included in this published article [and its supplementary information files].
