# Supplementary figures and images for: Phage–phage competition and biofilms affect interactions between two virulent bacteriophages and Pseudomonas aeruginosa
Source: ISME J. 2025 Apr 6;19(1):wraf065. doi: 10.1093/ismejo/wraf065 (PMC12041424; doi:10.1093/ismejo/wraf065)

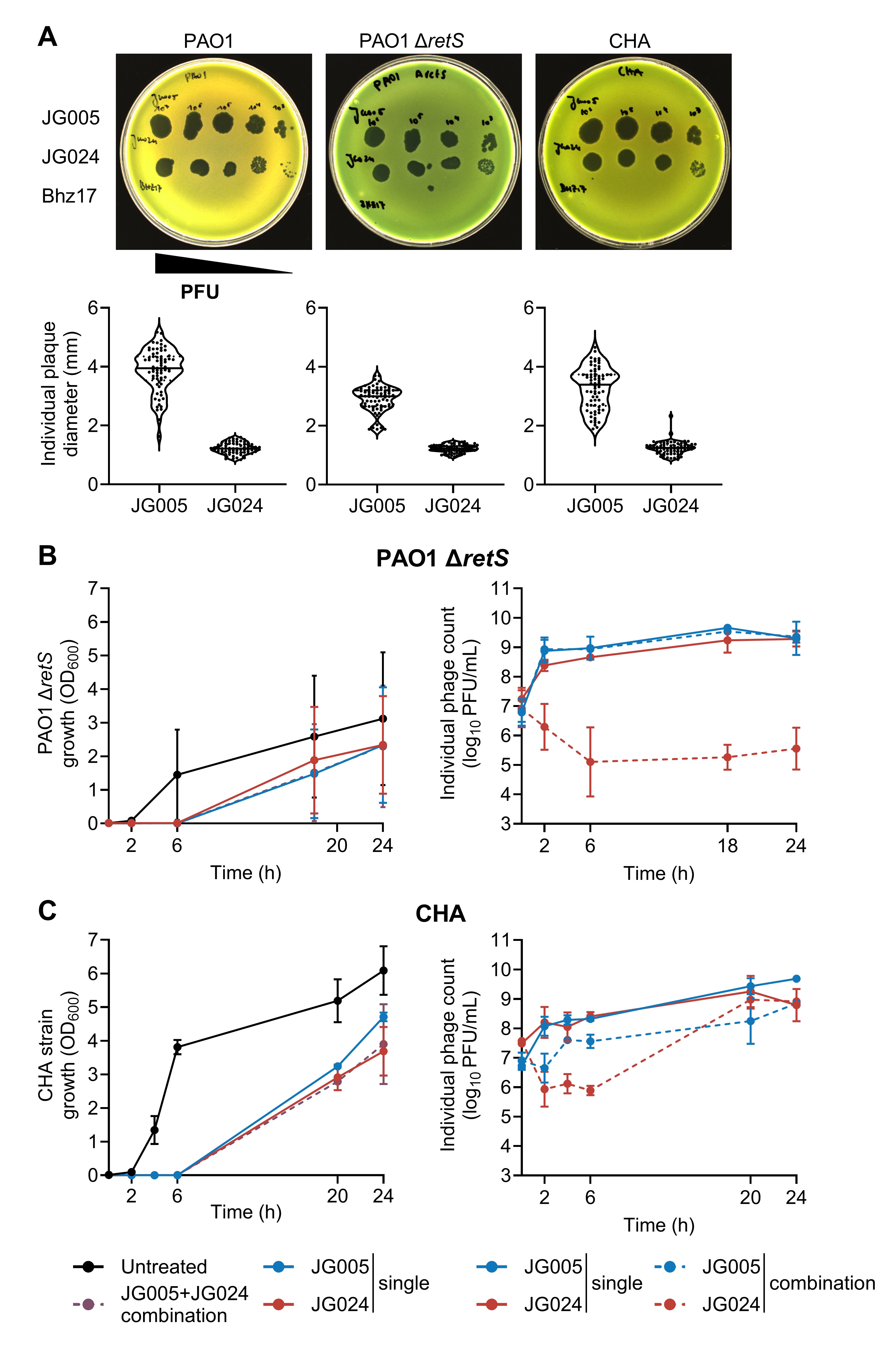

Supplement: Figure_S1_wraf065 [file figure_s1_wraf065.jpeg]

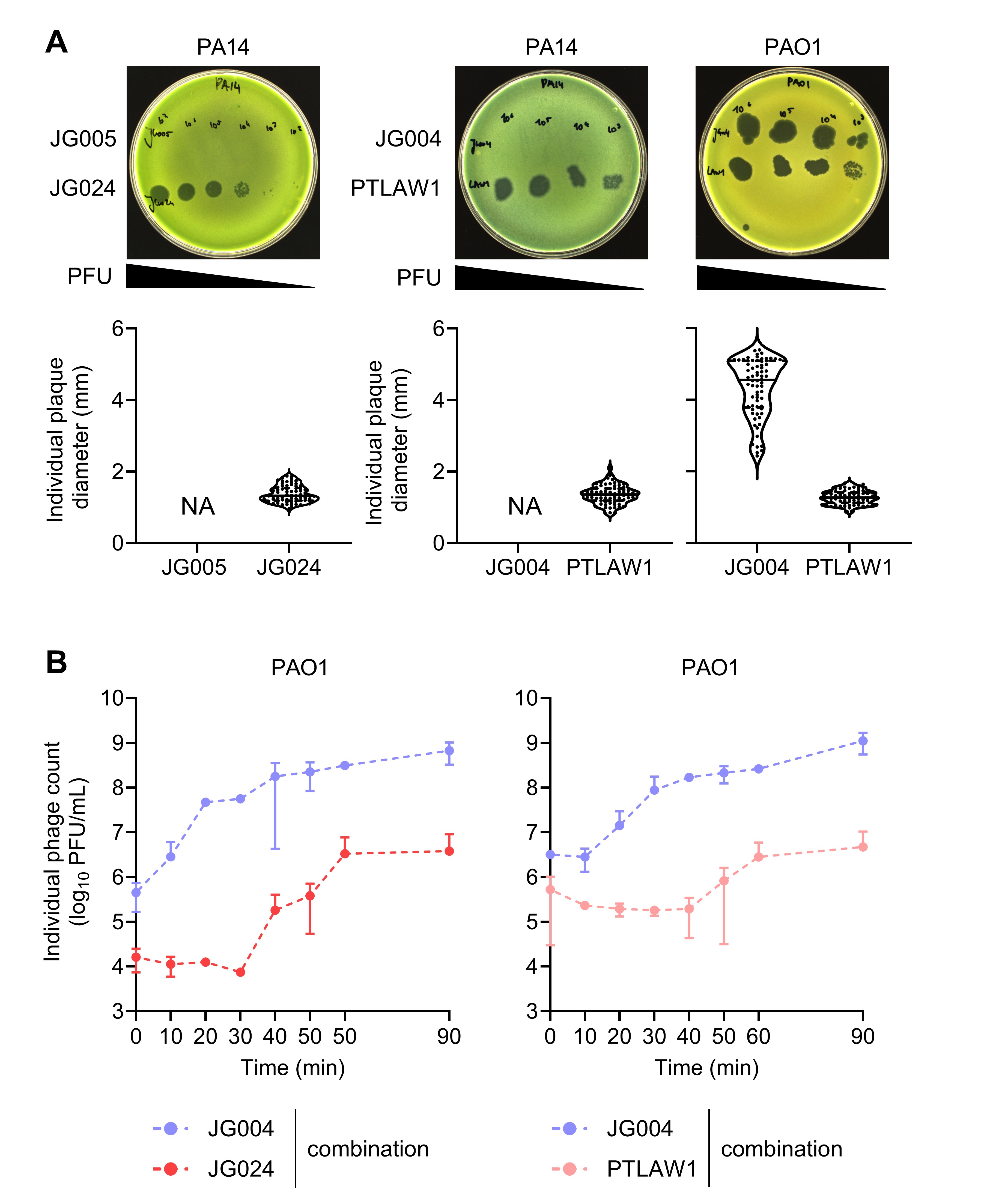

Supplement: Figure_S2_wraf065 [file figure_s2_wraf065.jpeg]

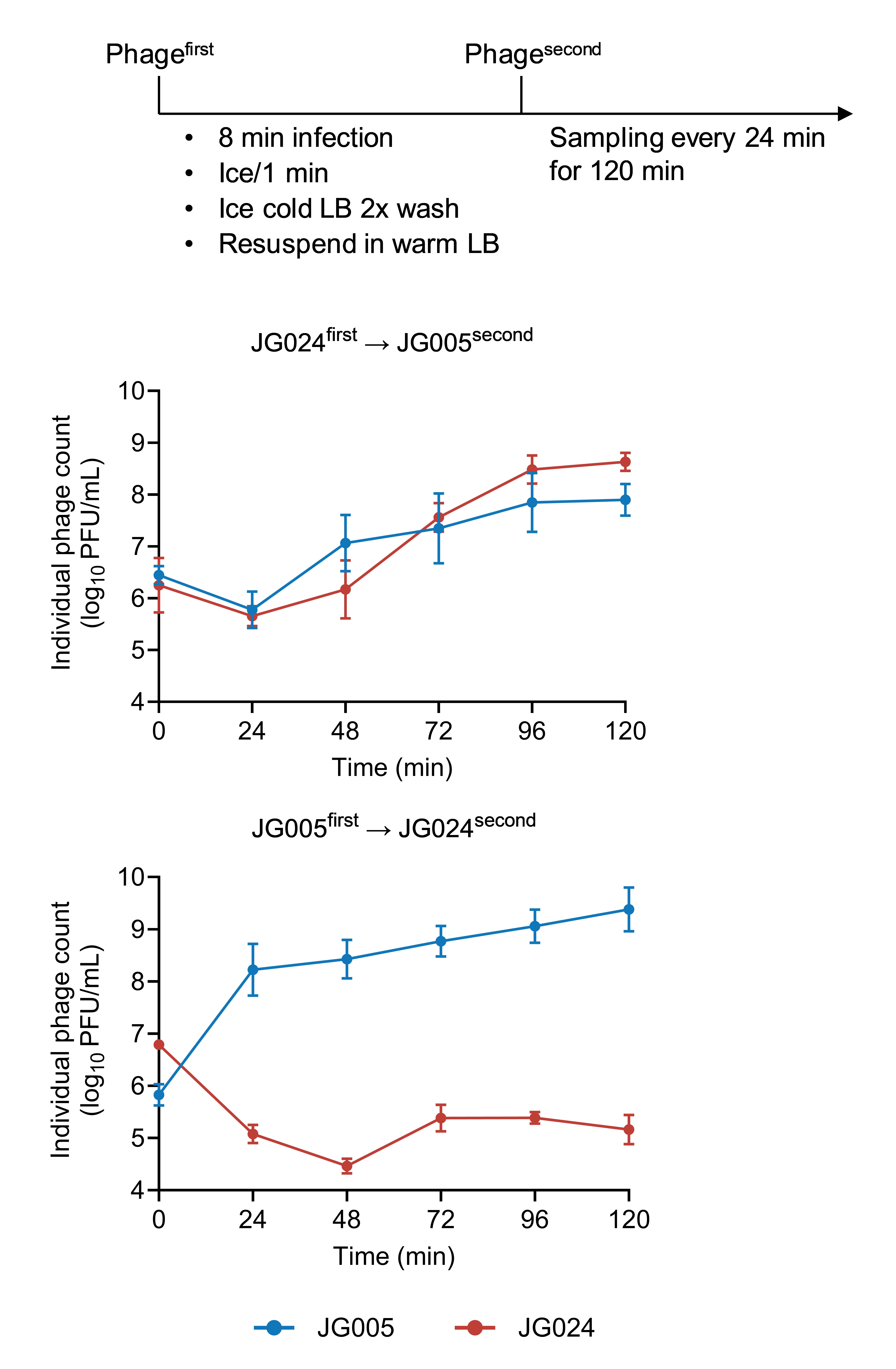

Supplement: Figure_S3_wraf065 [file figure_s3_wraf065.jpeg]

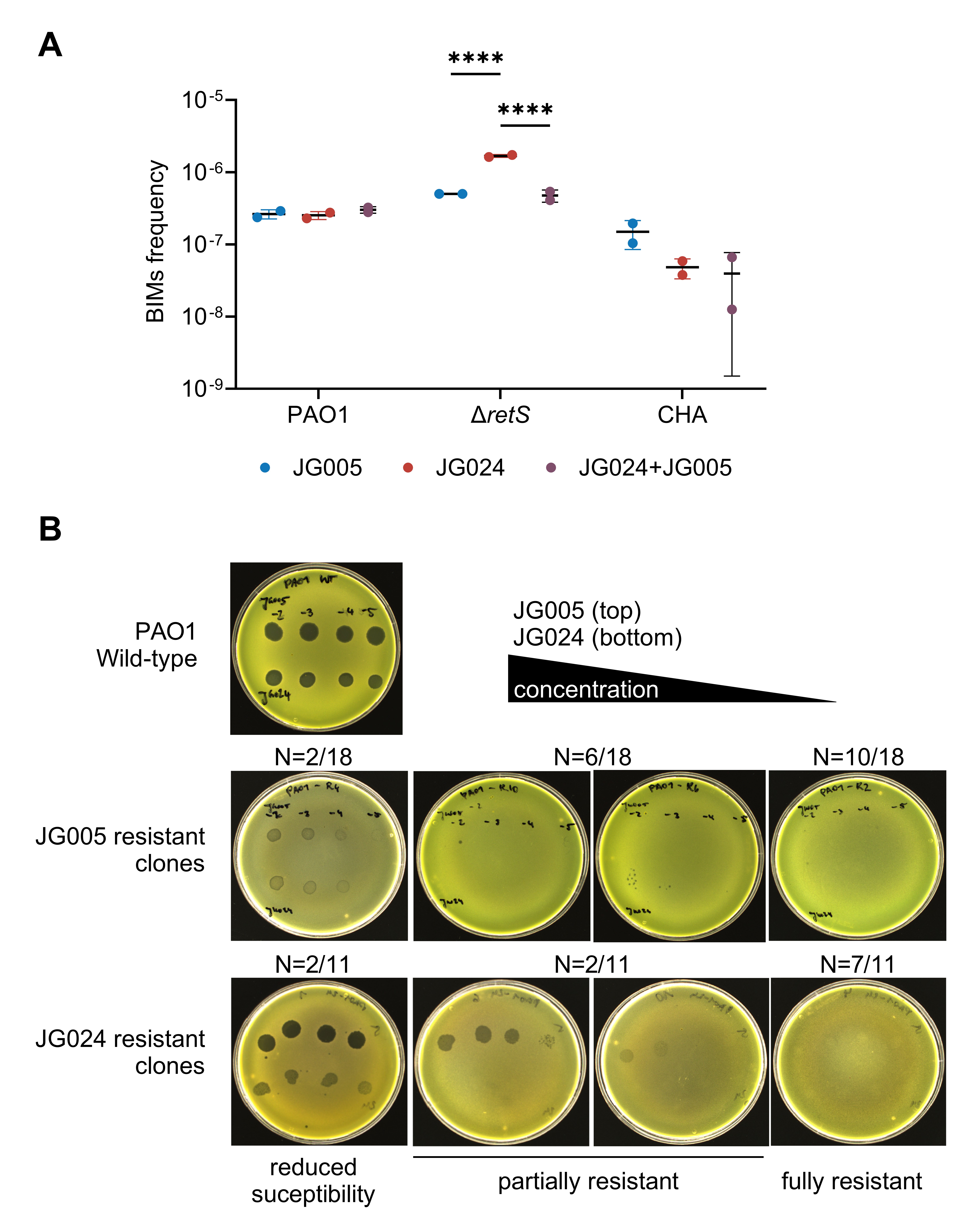

Supplement: Figure_S4_wraf065 [file figure_s4_wraf065.jpeg]
