# Supplementary material for: Phage–phage competition and biofilms affect interactions between two virulent bacteriophages and Pseudomonas aeruginosa
Source: ISME J. 2025 Apr 6;19(1):wraf065. doi: 10.1093/ismejo/wraf065 (PMC12041424; doi:10.1093/ismejo/wraf065)
Supplement: Supplementary_Table_S1_wraf065 [file supplementary_table_s1_wraf065.docx]

**Supplementary Table S1: Bacterial and phage isolates used in this study**

| **Bacterial strain and DSM number** | **Description** | **Source** |
| --- | --- | --- |
| *P. aeruginosa* PAO1  DSM 19880 | Wild-type strain/laboratory stock | DSMZ (Braunschweig, Germany) |
| *P.* *aeruginosa* PA14  DSM 19882 | Indicator strain for JG024 phage titration, and phage propagation |  |
| *P. aeruginosa* CH3549 **[1]**^a^  DSM 107571 | Indicator strain for Bhz17 phage titration, and phage propagation | TWINCORE, Centre for Clinical and Experimental Infection Research, (Hannover, Germany) and Molecular Bacteriology at Helmholtz Centre for Infection Research (Braunschweig, Germany) |
| *P. aeruginosa* F2230 **[1]**^b^ | Indicator strain for JG005 phage titration, and phage propagation |  |
| *P. aeruginosa* CHA **[2]** | Bronchopulmonary isolate from patient with Cystic Fibrosis | Jean-Marc Ghigo, Institut Pasteur (Paris, France) |
| *P. aeruginosa* PAO1 Δ*retS* **[3]** | Deletion mutant of *P. aeruginosa* PAO1 lacking *retS* gene |  |
| **Phage strain and DSM number** | **Characteristics** | **Source** |
| JG005  DSM 19872 | Class: *Caudoviricetes,* Genus: *Pakpunavirus*,  lytic on *P. aeruginosa* strains PAO1, CHA, F2230 (indicator strain). GenBank: PP712940.1 | DSMZ (Braunschweig, Germany) and Fraunhofer ITEM (Braunschweig, Germany) |
| JG024 **[4]**  DSM 22045 | Class: *Caudoviricetes,* Genus: *Pbunavirus*,  lytic on *P. aeruginosa* strains PAO1, PA14 (indicator strain). RefSeq: NC_017674.1 |  |
| Bhz17  DSM 107443 | Non-replicating phage on PAO1/PA14 strains. *P. aeruginosa* CH3549 is used as the indicator strain |  |
| JG004 **[5]**  DSM 19871 | Class: *Caudoviricetes,* Genus: *Pakpunavirus*,  lytic on *P. aeruginosa* strains PAO1, CHA, F2230 (indicator strain). RefSeq: NC_019450.1 | DSMZ (Braunschweig, Germany) |
| PTLAW1  DSM 105275 | Phylogenetically similar to JG024 phage (Imke H.E. Korf, unpublished data). Lytic on *P. aeruginosa* strains PAO1, PA14 (indicator strain). |  |
| ^a^ <https://bactome.helmholtz-hzi.de/cgi-bin/h-disol.cgi?STAT=4&Isol=CH3549> | | |
| ^b^ <https://bactome.helmholtz-hzi.de/cgi-bin/h-disol.cgi?STAT=4&Isol=F2230> | | |

**References**

1. Hornischer K, Khaledi A, Pohl S *et al.* Bactome—a reference database to explore the sequence-and gene expression-variation landscape of *pseudomonas aeruginosa* clinical isolates. *Nucleic Acids Res*. 2019;**47**:D716-D20 <https://doi.org/10.1093/nar/gky895>
2. Dacheux D, Toussaint B, Richard M *et al.* *Pseudomonas aeruginosa* cystic fibrosis isolates induce rapid, type iii secretion-dependent, but exou-independent, oncosis of macrophages and polymorphonuclear neutrophils. *Infect Immun*. 2000;**68**:2916-24 <https://doi.org/10.1128/iai.68.5.2916-2924.2000>
3. Goodman AL, Kulasekara B, Rietsch A *et al.* A signaling network reciprocally regulates genes associated with acute infection and chronic persistence in *pseudomonas aeruginosa*. *Dev Cell*. 2004;**7**:745-54 <https://doi.org/10.1016/j.devcel.2004.08.020>
4. Garbe J, Wesche A, Bunk B *et al.* Characterization of jg024, a *pseudomonas aeruginosa* pb1-like broad host range phage under simulated infection conditions. *BMC Microbiol*. 2010;**10**:1-10 <https://doi.org/10.1186/1471-2180-10-301>
5. Garbe J, Bunk B, Rohde M *et al.* Sequencing and characterization of *pseudomonas aeruginosa* phage jg004. *BMC Microbiol*. 2011;**11**:1-12 <https://doi.org/10.1186/1471-2180-11-102>
