# Supplementary material for: Phage–phage competition and biofilms affect interactions between two virulent bacteriophages and Pseudomonas aeruginosa
Source: ISME J. 2025 Apr 6;19(1):wraf065. doi: 10.1093/ismejo/wraf065 (PMC12041424; doi:10.1093/ismejo/wraf065)
Supplement: Supplementary_Table_S3_wraf065 [file supplementary_table_s3_wraf065.docx]

**Supplementary Table S3: Transcriptional responses of *P. aeruginosa* PAO1 and JG024 (with or without JG005 phage)**

**A. List of differentially expressed JG024 genes after infecting *P. aeruginosa* with JG024 alone or JG024+JG005 coinfection at 8 and 24 min.**

|  | **Single infection** | | **Co infection** | | **Gene function** |
| --- | --- | --- | --- | --- | --- |
| **JG024 Genes** | **8 min** | **24 min** | **8 min** | **24 min** |  |
| *F358_gp01* | 9.88502 | 10.19058 | 8.944853109 | 10.46459974 | hypothetical protein |
| *F358_gp02* | 7.235234 | 10.56562 | 6.603617119 | 11.02575452 | hypothetical protein |
| *F358_gp03* | 8.498931 | 10.11417 | 7.906706175 | 10.70384037 | hypothetical protein |
| *F358_gp04* | 7.072865 | 8.792465 | 6.041965719 | 8.61995135 | terminase large subunit |
| *F358_gp05* | 9.019505 | 9.413781 | 8.300293869 | 9.651846137 | hypothetical protein |
| *F358_gp06* | 8.288598 | 10.89185 | 7.770981024 | 11.16196446 | hypothetical protein |
| *F358_gp07* | 8.39224 | 13.13443 | 7.933932111 | 14.19998548 | hypothetical protein |
| *F358_gp08* | 8.102504 | 9.47713 | 7.452899849 | 9.728352325 | hypothetical protein |
| *F358_gp09* | 8.297202 | 9.542213 | 7.762517207 | 10.0523499 | hypothetical protein |
| *F358_gp10* | 9.233522 | 8.963824 | 8.813925658 | 9.208058654 | hypothetical protein |
| *F358_gp11* | 8.486995 | 9.150629 | 8.22039476 | 9.716011745 | hypothetical protein |
| *F358_gp12* | 8.621357 | 9.421578 | 7.984941247 | 9.734844608 | DUF6011 domain-containing protein |
| *F358_gp13* | 9.720861 | 7.647423 | 8.929218893 | 7.772825795 | methyltransferase |
| *F358_gp14* | 8.380937 | 9.630299 | 7.907108058 | 9.48019875 | hypothetical protein |
| *F358_gp15* | 9.664181 | 8.417874 | 8.927100582 | 9.021186112 | hypothetical protein |
| *F358_gp16* | 8.674024 | 8.952352 | 8.041207791 | 9.246621728 | hypothetical protein |
| *F358_gp17* | 7.905105 | 8.665016 | 7.608267809 | 9.403895412 | hypothetical protein |
| *F358_gp18* | 6.171186 | 8.610209 | 5.071067462 | 8.222210283 | minor head protein |
| *F358_gp19* | 5.575614 | 9.74482 | 4.088156073 | 9.691722581 | head morphogenesis |
| *F358_gp20* | 5.543286 | 7.861588 | 4.090685723 | 7.184739334 | hypothetical protein |
| *F358_gp21* | 5.896107 | 8.914975 | 0 | 9.602955646 | hypothetical protein |
| *F358_gp22* | 6.117686 | 9.09121 | 4.594330096 | 8.397254981 | head maturation protease |
| *F358_gp23* | 5.128195 | 8.748979 | 3.734915733 | 8.51881978 | virion structural protein |
| *F358_gp24* | 5.694169 | 9.517333 | 4.073759478 | 8.546590272 | major head protein |
| *F358_gp25* | 5.863934 | 8.684422 | 4.640042893 | 8.322231492 | hypothetical protein |
| *F358_gp26* | 5.209651 | 9.075454 | 3.650936886 | 8.556948224 | virion structural protein |
| *F358_gp27* | 5.319452 | 8.56859 | 3.742744579 | 8.657691235 | head protein |
| *F358_gp28* | 5.029611 | 8.162449 | 3.925022669 | 8.303631833 | hypothetical protein |
| *F358_gp29* | 6.710967 | 8.551947 | 5.439708008 | 8.484601051 | tail completion or Neck1 protein |
| *F358_gp30* | 6.027601 | 8.079865 | 4.779275909 | 7.527332293 | tail sheath |
| *F358_gp31* | 6.373126 | 8.729169 | 5.494501272 | 8.193510713 | tail fiber protein |
| *F358_gp32* | 6.264845 | 8.37977 | 5.088043689 | 7.83461549 | virion structural protein |
| *F358_gp33* | 5.720687 | 7.895612 | 4.684633788 | 7.416605438 | baseplate wedge subunit |
| *F358_gp34* | 4.692838 | 8.11165 | 3.527691298 | 7.893184611 | hypothetical protein |
| *F358_gp35* | 4.833584 | 8.282763 | 4.036984108 | 8.231314223 | tail fiber protein |
| *F358_gp36* | 5.653337 | 7.871235 | 4.715836793 | 8.182075067 | tail fiber protein |
| *F358_gp37* | 4.861141 | 7.074757 | 3.618928798 | 7.1961536 | tail fiber protein |
| *F358_gp38* | 7.950578 | 9.110603 | 7.141818466 | 9.220824614 | putative structural protein |
| *F358_gp39* | 6.167319 | 8.174427 | 5.298700924 | 7.947886978 | lytic tail protein |
| *F358_gp40* | 6.608238 | 8.27784 | 5.705598105 | 8.08035433 | putative lytic tail protein |
| *F358_gp41* | 5.894333 | 7.700541 | 5.267784502 | 7.456776351 | putative structural protein |
| *F358_gp42* | 6.825556 | 9.399097 | 5.876058342 | 9.180264219 | hypothetical protein |
| *F358_gp43* | 6.381357 | 8.514581 | 5.595534803 | 8.160599031 | baseplate spike |
| *F358_gp44* | 6.396046 | 8.326102 | 5.390716215 | 7.802351942 | baseplate wedge subunit |
| *F358_gp45* | 6.60718 | 9.047151 | 5.634132923 | 8.607402193 | DUF2612 domain-containing protein |
| *F358_gp46* | 6.307393 | 9.062201 | 5.512897345 | 8.629722215 | tail protein |
| *F358_gp47* | 6.780446 | 9.452732 | 5.730993757 | 8.746089928 | tail fiber protein |
| *F358_gp48* | 8.228578 | 9.540003 | 6.957265881 | 8.805187953 | putative endolysin |
| *F358_gp49* | 9.364613 | 9.526852 | 8.733545759 | 9.653909846 | endolysin |
| *F358_gp50* | 10.3801 | 9.260923 | 9.388255795 | 9.073176013 | putative DNA ligase |
| *F358_gp51* | 9.785157 | 9.05351 | 9.057684219 | 9.103739418 | MazG-like pyrophosphatase |
| *F358_gp52* | 10.01929 | 9.183702 | 9.048865867 | 9.443460413 | hypothetical protein |
| *F358_gp53* | 9.29999 | 9.273171 | 8.483355465 | 9.487695774 | hypothetical protein |
| *F358_gp54* | 9.141408 | 8.073043 | 8.260402903 | 7.785202757 | DNA helicase |
| *F358_gp55* | 9.747955 | 11.00263 | 8.954441619 | 11.12712184 | DNA helicase |
| *F358_gp56* | 10.18108 | 8.563154 | 9.412636249 | 8.313015835 | putative DNA helicase |
| *F358_gp57* | 9.841675 | 9.550742 | 8.905528795 | 9.412546115 | DNA polymerase |
| *F358_gp58* | 9.753888 | 8.999658 | 9.363233979 | 9.117047599 | 3'-5' exonuclease |
| *F358_gp59* | 11.82063 | 10.32143 | 11.52415831 | 11.43370435 | polynucleotide kinase/phosphorylase |
| *F358_gp60* | 8.80375 | 10.06127 | 8.30550881 | 10.12733745 | FAD-dependent thymidylate synthase |
| *F358_gp61* | 8.175235 | 9.701862 | 7.913627334 | 9.867508542 | putative thymidylate synthase |
| *F358_gp62* | 7.89421 | 9.509147 | 7.162708167 | 9.922267587 | hypothetical protein |
| *F358_gp63* | 8.972862 | 10.39212 | 7.860629109 | 10.21287609 | hypothetical protein |
| *F358_gp64* | 9.16939 | 8.99783 | 8.746854126 | 9.50465758 | hypothetical protein |
| *F358_gp65* | 8.296772 | 9.849061 | 7.858030623 | 10.3027524 | hypothetical protein |
| *F358_gp66* | 10.91669 | 9.038281 | 10.13264463 | 9.097287388 | hypothetical protein |
| *F358_gp67* | 9.623757 | 10.20068 | 8.411726154 | 9.706751931 | hypothetical protein |
| *F358_gp68* | 9.526271 | 8.865049 | 8.796011071 | 8.845486289 | putative structural protein |
| *F358_gp69* | 7.5602 | 10.20745 | 7.149019863 | 10.81642118 | Dda-like helicase |
| *F358_gp70* | 8.321961 | 10.1168 | 7.627314719 | 10.38420471 | hypothetical protein |
| *F358_gp71* | 8.73723 | 9.351395 | 8.3089932 | 9.617007836 | hypothetical protein |
| *F358_gp72* | 8.732401 | 10.00833 | 8.952332094 | 10.25701431 | hypothetical protein |
| *F358_gp73* | 9.121518 | 10.72297 | 8.61932017 | 10.63668286 | hypothetical protein |
| *F358_gp74* | 8.460705 | 8.759752 | 8.249367078 | 8.421562548 | DNA primase |
| *F358_gp75* | 6.90198 | 10.27043 | 7.142248625 | 10.47981252 | hypothetical protein |
| *F358_gp76* | 8.560232 | 9.143215 | 8.135412079 | 8.846583286 | DNA primase |
| *F358_gp77* | 7.646614 | 7.433033 | 7.326406068 | 6.951930925 | putative primase |
| *F358_gp78* | 6.046271 | 9.818073 | 5.258106049 | 10.11889684 | hypothetical protein |
| *F358_gp79* | 7.536956 | 9.44205 | 6.376551672 | 9.229204672 | hypothetical protein |
| *F358_gp80* | 8.622134 | 9.539246 | 8.035220553 | 9.922900662 | hypothetical protein |
| *F358_gp81* | 8.570569 | 9.965725 | 8.467374124 | 10.29203059 | hypothetical protein |
| *F358_gp82* | 9.262597 | 8.23136 | 8.599353386 | 8.908058121 | hypothetical protein |
| *F358_gp83* | 9.149264 | 9.851397 | 8.666029985 | 10.48513111 | hypothetical protein |
| *F358_gp84* | 9.063609 | 9.651643 | 8.38990714 | 9.89248667 | hypothetical protein |
| *F358_gp85* | 8.3883 | 8.714492 | 7.871302244 | 9.347919306 | hypothetical protein |
| *F358_gp86* | 10.39332 | 12.45291 | 9.877722667 | 13.22106104 | hypothetical protein |
| *F358_gp87* | 8.561256 | 9.873819 | 8.35634598 | 10.35173961 | hypothetical protein |
| *F358_gp88* | 3.729052 | 10.20931 | 0 | 10.3416466 | hypothetical protein |
| *F358_gp89* | 8.900974 | 9.342467 | 8.309002174 | 9.86231578 | hypothetical protein |
| *F358_gp90* | 8.931785 | 10.32856 | 7.963890588 | 10.57328781 | hypothetical protein |
| *F358_gp91* | 8.156214 | 9.588406 | 7.524255594 | 9.782128371 | hypothetical protein |
| *F358_gp92* | 7.788457 | 9.083352 | 7.465035394 | 9.060418793 | hypothetical protein |
| *F358_gp93* | 8.562082 | 9.964831 | 8.170871087 | 10.30603644 | tail length tape measure protein |

**B. List of differentially expressed JG005 genes after co-infection with JG024 in *P. aeruginosa* PAO1 at 8 and 24 min.**

| **JG005 genes** | **8 min** | **24 min** | **Gene function** |
| --- | --- | --- | --- |
| *JG005_003* | 11.55175699 | 14.06810549 | hypothetical protein |
| *JG005_004* | 10.35974895 | 12.40567295 | hypothetical protein |
| *JG005_005* | 10.65758734 | 10.33439753 | hypothetical protein |
| *JG005_006* | 10.11893857 | 14.95523306 | hypothetical protein |
| *JG005_007* | 8.326445968 | 13.61418568 | hypothetical protein |
| *JG005_008* | 12.40544887 | 12.51753591 | hypothetical protein |
| *JG005_009* | 9.01342084 | 10.819322 | hypothetical protein |
| *JG005_010* | 8.286593349 | 11.550023 | hypothetical protein |
| *JG005_011* | 9.821777646 | 11.31450781 | hypothetical protein |
| *JG005_013* | 9.513476094 | 10.22456333 | hypothetical protein |
| *JG005_014* | 10.28578452 | 11.56276846 | hypothetical protein |
| *JG005_015* | 9.460811547 | 10.98639879 | hypothetical protein |
| *JG005_016* | 9.79072998 | 15.27736969 | hypothetical protein |
| *JG005_017* | 9.35735808 | 10.8494775 | hypothetical protein |
| *JG005_018* | 10.14355437 | 11.59886564 | hypothetical protein |
| *JG005_019* | 9.798533715 | 6.322664391 | hypothetical protein |
| *JG005_020* | 9.539432781 | 11.4566784 | hypothetical protein |
| *JG005_021* | 12.28560406 | 9.346936436 | hypothetical protein |
| *JG005_022* | 9.913533687 | 10.62516283 | hypothetical protein |
| *JG005_023* | 8.905946307 | 10.999903 | hypothetical protein |
| *JG005_024* | 8.760380981 | 13.44881299 | hypothetical protein |
| *JG005_025* | 10.33694645 | 12.47286665 | hypothetical protein |
| *JG005_026* | 9.886271264 | 9.668062421 | hypothetical protein |
| *JG005_027* | 10.27232409 | 11.5800384 | hypothetical protein |
| *JG005_028* | 9.246677974 | 12.28736016 | hypothetical protein |
| *JG005_029* | 11.06047486 | 14.13252128 | hypothetical protein |
| *JG005_030* | 10.5169413 | 13.79838441 | hypothetical protein |
| *JG005_031* | 8.826250837 | 10.30940402 | hypothetical protein |
| *JG005_032* | 5.826544461 | 3.972850165 | hypothetical protein |
| *JG005_033* | 12.95143533 | 9.919593521 | hypothetical protein |
| *JG005_034* | 9.14333412 | 10.04663514 | hypothetical protein |
| *JG005_035* | 9.894235832 | 14.09900447 | hypothetical protein |
| *JG005_036* | 11.69650963 | 13.95941838 | hypothetical protein |
| *JG005_037* | 11.96689695 | 10.73660987 | hypothetical protein |
| *JG005_038* | 8.887033039 | 11.84465004 | hypothetical protein |
| *JG005_039* | 9.574264942 | 10.16310251 | hypothetical protein |
| *JG005_040* | 12.39291527 | 12.77016438 | hypothetical protein |
| *JG005_041* | 9.918383568 | 13.45221744 | hypothetical protein |
| *JG005_042* | 10.09538884 | 11.19402984 | hypothetical protein |
| *JG005_043* | 10.30562691 | 12.67830717 | hypothetical protein |
| *JG005_044* | 10.64768631 | 9.861642896 | Ribonucleotide reductase |
| *JG005_045* | 10.31894423 | 10.35112333 | Ribonucleotide reductase |
| *JG005_046* | 10.31022001 | 10.99864716 | hypothetical protein |
| *JG005_047* | 9.769735048 | 9.650928677 | Thymidylate synthase |
| *JG005_048* | 11.17234989 | 10.16464207 | hypothetical protein |
| *JG005_049* | 9.002911348 | 12.09559624 | hypothetical protein |
| *JG005_050* | 10.89247745 | 11.38024597 | hypothetical protein |
| *JG005_051* | 10.29889885 | 12.44139398 | hypothetical protein |
| *JG005_054* | 10.63730218 | 15.36490855 | hypothetical protein |
| *JG005_055* | 10.40915393 | 10.63629467 | hypothetical protein |
| *JG005_056* | 11.40781216 | 14.52761026 | hypothetical protein |
| *JG005_057* | 9.861751989 | 13.86988859 | hypothetical protein |
| *JG005_058* | 10.83697912 | 10.95904278 | HNH nuclease |
| *JG005_059* | 9.45032129 | 15.04265297 | hypothetical protein |
| *JG005_060* | 10.6398653 | 12.05201682 | hypothetical protein |
| *JG005_061* | 10.1988838 | 12.09947291 | hypothetical protein |
| *JG005_062* | 9.517916048 | 10.31105614 | hypothetical protein |
| *JG005_063* | 10.15892207 | 11.70993198 | hypothetical protein |
| *JG005_064* | 9.202188021 | 12.64045198 | hypothetical protein |
| *JG005_065* | 10.12604574 | 10.62107464 | DNA polymerase |
| *JG005_066* | 10.15632836 | 9.769526554 | DNA primase/helicase |
| *JG005_067* | 9.420776675 | 14.56927517 | hypothetical protein |
| *JG005_068* | 9.796155106 | 10.8536753 | hypothetical protein |
| *JG005_069* | 9.661363251 | 10.65927856 | hypothetical protein |
| *JG005_070* | 10.57513035 | 11.53138132 | hypothetical protein |
| *JG005_071* | 9.587076048 | 11.43077205 | hypothetical protein |
| *JG005_072* | 10.45000303 | 10.0130132 | hypothetical protein |
| *JG005_073* | 9.581436426 | 9.279782247 | hypothetical protein |
| *JG005_074* | 10.48853191 | 12.23413471 | hypothetical protein |
| *JG005_075* | 10.17224994 | 11.86361931 | hypothetical protein |
| *JG005_076* | 11.16554388 | 11.59932532 | hypothetical protein |
| *JG005_077* | 9.416432185 | 10.72113712 | hypothetical protein |
| *JG005_078* | 11.23890859 | 11.38262804 | hypothetical protein |
| *JG005_079* | 10.42966874 | 9.533183033 | hypothetical protein |
| *JG005_080* | 10.04219881 | 9.535166392 | hypothetical protein |
| *JG005_081* | 10.5402638 | 10.72697315 | hypothetical protein |
| *JG005_082* | 9.430062989 | 10.5293204 | RNA ligase |
| *JG005_083* | 11.03262425 | 8.114585993 | hypothetical protein |
| *JG005_084* | 9.969586334 | 10.6066589 | hypothetical protein |
| *JG005_085* | 11.76100601 | 9.465833494 | hypothetical protein |
| *JG005_086* | 7.660705109 | 6.250100837 | hypothetical protein |
| *JG005_087* | 9.867997145 | 11.31217711 | hypothetical protein |
| *JG005_088* | 10.26894106 | 10.09285414 | hypothetical protein |
| *JG005_089* | 10.75358971 | 12.879814 | hypothetical protein |
| *JG005_090* | 4.967067326 | 8.696649039 | hypothetical protein |
| *JG005_091* | 7.645324493 | 8.312167068 | hypothetical protein |
| *JG005_092* | 7.378106958 | 11.35897527 | Tail fiber assembly protein |
| *JG005_093* | 6.700426069 | 15.2186901 | Tail fiber assembly protein |
| *JG005_094* | 11.3528475 | 14.03287001 | Tail fiber assembly protein |
| *JG005_095* | 8.268859166 | 11.4250101 | hypothetical protein |
| *JG005_096* | 8.618404107 | 12.24943054 | Baseplate components |
| *JG005_097* | 7.454062125 | 10.9724234 | hypothetical protein |
| *JG005_098* | 8.712602576 | 10.79770366 | hypothetical protein |
| *JG005_099* | 9.697609801 | 12.03647038 | hypothetical protein |
| *JG005_100* | 9.027742931 | 12.09487156 | hypothetical protein |
| *JG005_101* | 7.555439789 | 10.16864961 | hypothetical protein |
| *JG005_102* | 8.21103846 | 13.14869299 | Tape measure protein |
| *JG005_103* | 8.424078257 | 11.00132834 | hypothetical protein |
| *JG005_104* | 6.388056896 | 11.37834063 | hypothetical protein |
| *JG005_105* | 8.815536783 | 10.8322975 | hypothetical protein |
| *JG005_106* | 7.470430829 | 8.373996698 | hypothetical protein |
| *JG005_107* | 11.98763477 | 13.72864758 | hypothetical protein |
| *JG005_108* | 9.49805305 | 15.16861978 | Endonuclease |
| *JG005_109* | 9.534417747 | 15.65902944 | Structural protein |
| *JG005_110* | 8.042807514 | 15.65087436 | hypothetical protein |
| *JG005_111* | 8.25189352 | 12.05499343 | hypothetical protein |
| *JG005_112* | 9.175881616 | 11.57340908 | RNA polymerase |
| *JG005_113* | 7.573164388 | 12.32660594 | hypothetical protein |
| *JG005_114* | 7.669215625 | 12.45901026 | Major capsid protein |
| *JG005_115* | 8.498759649 | 11.65522302 | hypothetical protein |
| *JG005_116* | 7.103257438 | 12.35051333 | hypothetical protein |
| *JG005_117* | 8.705153909 | 12.92930315 | Methytransferase |
| *JG005_118* | 9.088788476 | 11.65002472 | hypothetical protein |
| *JG005_119* | 6.880231017 | 11.40040937 | Terminase |
| *JG005_134* | 8.54828018 | 12.57388141 | hypothetical protein |
| *JG005_136* | 8.843136119 | 11.02687391 | hypothetical protein |
| *JG005_137* | 9.551068032 | 11.69837004 | hypothetical protein |
| *JG005_138* | 9.804814713 | 11.66976236 | hypothetical protein |
| *JG005_139* | 9.676333352 | 15.44517356 | hypothetical protein |
| *JG005_140* | 11.04486914 | 10.72781673 | hypothetical protein |
| *JG005_141* | 11.98431454 | 12.29686851 | hypothetical protein |
| *JG005_142* | 10.70819491 | 10.94809021 | hypothetical protein |
| *JG005_143* | 10.91469864 | 11.5307284 | hypothetical protein |
| *JG005_144* | 11.47628502 | 10.93930634 | hypothetical protein |
| *JG005_145* | 9.901976861 | 15.24518835 | hypothetical protein |
| *JG005_146* | 9.852394254 | 13.06332383 | hypothetical protein |
| *JG005_147* | 9.937882931 | 10.83541997 | hypothetical protein |
| *JG005_148* | 10.58748804 | 9.928256984 | hypothetical protein |
| *JG005_149* | 10.27957938 | 10.17283245 | hypothetical protein |
| *JG005_150* | 11.38700245 | 10.41325403 | hypothetical protein |
| *JG005_151* | 10.4270417 | 11.15270172 | hypothetical protein |
| *JG005_152* | 12.32260601 | 9.811326078 | hypothetical protein |
| *JG005_153* | 11.26776887 | 9.164654 | hypothetical protein |
| *JG005_154* | 10.18462635 | 13.69699603 | hypothetical protein |
| *JG005_155* | 10.20400317 | 11.06570171 | Protease subunit |
| *JG005_156* | 10.80053341 | 14.75083648 | hypothetical protein |
| *JG005_157* | 11.83410291 | 9.718815669 | hypothetical protein |
| *JG005_158* | 10.50667188 | 10.52833115 | hypothetical protein |
| *JG005_159* | 10.45092836 | 9.027558412 | hypothetical protein |
| *JG005_160* | 9.795207584 | 10.8715971 | hypothetical protein |
| *JG005_161* | 10.78134977 | 12.06391446 | endonuclease |
| *JG005_162* | 11.02544312 | 11.06317378 | hypothetical protein |
| *JG005_163* | 9.823022303 | 12.17160679 | hypothetical protein |
| *JG005_164* | 10.86989926 | 11.87515639 | hypothetical protein |
| *JG005_165* | 10.39373558 | 11.75253899 | dCMP deaminase |
| *JG005_166* | 10.69416232 | 11.89144165 | DNA ligase |
| *JG005_167* | 10.52979452 | 13.5941982 | hypothetical protein |
| *JG005_168* | 10.58155558 | 10.34552695 | Cell wall hydrolase |
| *JG005_169* | 10.81417715 | 12.51359996 | Phospho hydrolase |
| *JG005_170* | 11.64527849 | 14.03480318 | Phospho esterase |
| *JG005_171* | 10.20410463 | 11.08446758 | hypothetical protein |
| *JG005_172* | 12.43391313 | 11.29653632 | hypothetical protein |
| *JG005_173* | 9.39031777 | 12.38374894 | hypothetical protein |
| *JG005_174* | 9.310129995 | 10.55630387 | hypothetical protein |
| *JG005_175* | 10.54891023 | 12.31713266 | RNA ligase |
| *JG005_176* | 11.348436 | 10.11721541 | hypothetical protein |
| *JG005_177* | 9.896603038 | 10.97008545 | Ribose-phosphate pyrophosphokinase |
| *JG005_178* | 14.32855606 | 10.98910292 |  |
| *JG005_179* | 9.708859829 | 14.24539396 | Nictotinate phosphoribosyltransferase |
| *JG005_180* | 11.09649628 | 10.63866672 | hypothetical protein |
| *JG005_181* | 10.59416446 | 11.76790101 | hypothetical protein |
| *JG005_182* | 10.48283557 | 13.62076207 | hypothetical protein |
| *JG005_183* | 11.40367946 | 11.94430665 | hypothetical protein |
| *JG005_184* | 10.36322446 | 11.18131041 | hypothetical protein |
| *JG005_185* | 5.682889023 |  | hypothetical protein |
| *JG005_186* | 8.713167631 | 8.78449288 | hypothetical protein |

**C. List of differentially expressed *P. aeruginosa* PAO1 genes after mono- or co-infection with JG024 and JG024+JG005 phages at 8 and 24 min**

Several of the differentially expressed genes under JG024 and/or JG025+JG005 infection conditions are related to cell wall modification, transporters, nutrient acquisition system, global stress responses, two-component regulatory systems and replication, transcription and translation processes. Many of these gene expression patterns are similar to those found in *P. aeruginosa* infected with PAK_P4, Pseudomonas phage ɸKZ, PA5oct and LUZ19 (Brandão et al. 2021 Wicke et al. 2021 Lood et al. 2020 and Chevallereau et al. 2016) Although the duration of infection, bacterial strain type, stirred growth condition and bacterial growth medium contribute to a small set of unique phage-specific transcriptional signatures, a greater convergence in the transcriptional response indicates a global stress response of *P. aeruginosa* to dsDNA phages.

- Brandão A, Pires DP, Coppens L *et al.* Differential transcription profiling of the phage luz19 infection process in different growth media. *RNA biology*. 2021;**18**:1778-90
- Wicke L, Ponath F, Coppens L *et al.* Introducing differential rna-seq mapping to track the early infection phase for pseudomonas phage ɸkz. *RNA biology*. 2021;**18**:1099-110
- Lood C, Danis‐Wlodarczyk K, Blasdel BG *et al.* Integrative omics analysis of pseudomonas aeruginosa virus pa5oct highlights the molecular complexity of jumbo phages. *Environmental Microbiology*. 2020;**22**:2165-81
- Chevallereau A, Blasdel BG, De Smet J *et al.* Next-generation “-omics” approaches reveal a massive alteration of host rna metabolism during bacteriophage infection of *pseudomonas aeruginosa*. *PLoS Genet*. 2016;**12**:e1006134
- Macé C, Seyer D, Chemani C, Cosette P, Di-Martino P, Guery B, Filloux A, Fontaine M, Molle V, Junter GA, Jouenne T. Identification of biofilm-associated cluster (bac) in *Pseudomonas aeruginosa* involved in biofilm formation and virulence. PLoS One. 2008;3(12):e3897. (listed below)
- Bonneau A, Roche B, Schalk IJ. Iron acquisition in Pseudomonas aeruginosa by the siderophore pyoverdine: an intricate interacting network including periplasmic and membrane proteins. Sci Rep. 2020 Jan 10;10(1):120. (listed below)

As illustrated in the following tables, the differentially expressed genes are listed according to each condition that was tested. In view of the substantial overlap between this data and that from other studies, the genes associated with cell wall modification, transporters, nutrient acquisition systems, global stress responses, two-component regulatory systems and replication, transcription and translation processes are not highlighted.

However, some other notable genes with a similar transcriptional pattern that have been observed in other studies are emphasized.

| **Differentially expressed *P. aeruginosa* genes in at least three tested conditions at 8 and 24 minutes.** | | | | | | |
| --- | --- | --- | --- | --- | --- | --- |
|  | **8 minutes** | | **24 minutes** | |  |  |
|  | **JG024+JG005** | **JG024** | **JG005+JG024** | **JG024** | **Gene function** | **Similar observation in other studies** |
| *aprE* | -1.74225624 | -7.07265186 | -2.774191208 | -1.894079816 | alkaline protease secretion protein AprE |  |
| *aprI* | -1.594035064 | -1.922126691 | -1.643948862 | -1.774782033 | alkaline proteinase inhibitor AprI |  |
| *bfrB* | 1.555513938 | 1.999533754 | 1.896316959 | 1.532326485 | Bacterioferritin (cytochrome b1) | Wicke et al. 2021 |
| *gcdH* | 2.706842185 | 2.431230219 | 1.736486042 | 2.012389626 | glutaryl-CoA dehydrogenase |  |
| *hmgA* | 1.99046888 |  | 2.201593664 | 2.004307183 | homogentisate 1,2-dioxygenase |  |
| *hpd* | 1.774800026 |  | 2.881874426 | 2.403180725 | 4-hydroxyphenylpyruvate dioxygenase and related hemolysins |  |
| *lecA* | 3.524632926 | 2.948737301 | 5.347644618 | 4.24902957 | PA-I galactophilic lectin |  |
| *nirF* |  | -1.769752634 | -1.733161119 | -1.954557025 | heme d1 biosynthesis protein NirF |  |
| *nirN* |  | -1.508414388 | -1.967148387 | -2.194872495 | Cytochrome D1 heme domain |  |
| *PA0526* |  | -1.511026908 | -1.810259094 | -1.975378372 |  |  |
| *PA0547* |  | 1.644098239 | 2.064041228 | 2.022323926 |  |  |
| *PA0713* |  | -1.796716741 | -2.415574264 | -1.879147868 |  |  |
| *PA0805* | 1.730416558 | 2.333350357 | 2.001301873 | 1.815057752 | (LasR binding site predicted) |  |
| *PA1692* | -1.696811399 |  | -1.830660833 | -1.581956158 | probable translocation protein in type III secretion | Wicke et al. 2021. and Brandão et al. 2020 |
| *PA2045* |  | 2.108209534 | 1.526695423 | 1.852035118 |  |  |
| *PA2485* | 1.7901575 | 1.99477605 | 4.262557622 | 3.371887736 |  |  |
| *PA2826* | 3.013328588 | 3.137181971 | 2.440936607 | 1.852193369 | Glutathione peroxidase, house-cleaning role in reducing lipid peroxides |  |
| *PA3728* |  | 1.67041438 | 1.794696699 | 1.635326836 | swarm and to produce extracellular rhamnolipids, phage shock proteins, | Macé et al. 2008 |
| *PA3729* | 2.547730453 | 2.797545084 | 1.905526301 | 1.641697788 |  |  |
| *PA3730* | 2.270786034 | 2.767148223 | 2.542035672 | 2.138774118 |  |  |
| *PA3731* | 2.234898053 | 2.951944756 | 2.816367945 | 2.991447833 |  |  |
| *PA3732* | 3.054681066 | 3.242660384 | 2.443809058 | 2.067059804 |  |  |
| *PA3911* | -2.305687738 | -2.821937763 | -1.698958502 | -2.989356029 | Predicted lipid carrier protein YhbT |  |
| *PA4063* | 2.488898759 | 2.730337821 | 2.992448937 | 2.281644136 |  |  |
| *PA4364* | 2.993215216 | 2.621496871 | 3.112909168 | 2.045369388 |  |  |
| *PA4365* | 2.267703778 | 2.048509677 | 2.537083209 | 1.548900622 | Arginine exporter |  |
| *PA4582* |  | 1.516645909 | 2.206750943 | 1.882824212 |  |  |
| *PA4610* | -2.23658811 | -1.972289026 | -1.851777872 | -1.640028077 |  |  |
| *PA4624* | 2.202100541 | 1.97762557 | 2.247645382 | 1.660337383 | Hemolysin activation/secretion protein | Wicke et al. 2021 and Brandão et al. 2020 |
| *PA4625* | 2.933896253 | 2.731048961 | 1.936793525 | 1.590504497 | cyclic diguanylate-regulated TPS partner A, CdrA | Wicke et al. 2021 |
| *PA5440* | -1.983494148 |  | -1.826319572 | -1.649110831 | Sulfur transporter |  |

**Unique subset of differentially expressed *P. aeruginosa* genes during JG024 or JG024+JG005 co-infection at 8 minutes (these expression levels were not statistically at 24 minutes).**

| **Differentially expressed *P. aeruginosa* genes only during JG024 single or JG024+JG005 co-infection at 8 minutes** | | | | |
| --- | --- | --- | --- | --- |
|  | **JG024+JG005** | **JG024** | **Gene function** | **Similar observation in other studies** |
| *capB* | 1.737080167 | 2.494817314 | Cold shock proteins |  |
| *dnaK* | 1.509952086 | 2.118134621 |  |  |
| *fumC1* | 1.949593628 | 2.205225377 | fumarate hydratase |  |
| *gbcA* | -2.446772564 | -1.94140955 | glycine betaine catabolism protein |  |
| *htpG* | 1.535140404 | 2.424430041 | Posttranslational modification |  |
| *ibpA* | 2.067685928 | 2.573967599 | heat-shock protein IbpA |  |
| *narI* | -1.690677966 | -2.172175678 | respiratory nitrate reductase gamma chain |  |
| *narK1* | -2.052738953 | -2.063775117 | nitrite extrusion protein 1 |  |
| *PA0573* | -1.89246487 | -1.517656848 |  |  |
| *PA0634* | 1.617530823 | 2.039607375 | Phage tail assembly chaperone |  |
| *PA0779* | 1.804019135 | 2.300813114 | ATP-dependent Lon protease, bacterial type |  |
| *PA0781* | 2.325044261 | 2.330707093 |  |  |
| *PA1597* | 2.224428228 | 2.996729962 | Dienelactone hydrolase |  |
| *PA1848* | -1.755565843 | -2.663593623 |  |  |
| *PA2179* | -2.146101043 | -1.72179639 |  |  |
| *PA2501* | -2.035481461 | -1.532884308 |  |  |
| *PA2602* | 1.929267501 | 1.823809831 |  |  |
| *PA2753* | -1.643097506 | -1.712776708 |  |  |
| *PA2939* | 2.07690862 | 2.738795888 | probable aminopeptidase |  |
| *PA3205* | 1.678278995 | 1.894624537 | Periplasmic protein refolding chaperone Spy/CpxP family |  |
| *PA3432* | -1.927031759 | -1.943011446 |  |  |
| *PA3598* | 1.526949 | 2.141120694 |  |  |
| *PA3691* | 1.812457823 | 2.139815933 |  |  |
| *PA4141* | 1.692426131 | 2.305699531 |  |  |
| *PA4515* | -1.546727601 | -1.546420874 | Fe(II)-dependent oxygenase superfamily protein; |  |
| *PA4516* | -1.607540168 | -1.842196401 |  |  |
| *PA5181* | 1.878968843 | 1.725714967 | oxidoreductase alpha (molybdopterin) subunit |  |
| *pmtA* | 2.832247372 | 2.592982402 | phospholipid methyltransferase |  |
| **Differentially expressed *P. aeruginosa* genes only during JG024 single infection at 8 minutes** | | | | |
|  | **JG024+JG005** | **JG024** | **Gene function** | **Similar observation in other studies** |
| *aotM* |  | -1.568465014 | arginine/ornithine transport protein AotM |  |
| *chpE* |  | -1.520459944 | probable chemotaxis protein |  |
| *cupB1* |  | 1.863999795 | fimbrial subunit |  |
| *cupC2* |  | 2.330257946 | fimbrial subunit / pilus subunit |  |
| *gapA* |  | 1.539077122 | glyceraldehyde 3-phosphate dehydrogenase |  |
| *groEL* |  | 1.693926665 | Chaperone groEL |  |
| *hpaC* |  | 3.402164549 | 4-hydroxyphenylacetate 3-monooxygenase small chain |  |
| *hpcD* |  | 3.873375036 | 5-carboxymethyl-2-hydroxymuconate isomerase |  |
| *hslU* |  | 1.833345691 | heat shock protein HslU |  |
| *hslV* |  | 1.796412039 | heat shock protein HslV |  |
| *moaA1* |  | -1.768891464 | Mo cofactor biosynthesis |  |
| *moeA1* |  | -1.665624116 | Mo cofactor biosynthesis |  |
| *nirB* |  | 1.675352946 | assimilatory nitrite reductase large subunit |  |
| *nirC* |  | -1.580099999 | probable c-type cytochrome precursor |  |
| *nirL* |  | -2.252345983 | heme biosynthesis protein |  |
| *nosF* |  | -1.601381777 | nitrous oxide reductase maturation ATPase. copper binding protein |  |
| *nosL* |  | -1.769412476 | copper binding lipoprotein |  |
| *oprI* |  | 1.508900487 | outer membrane lipoprotein |  |
| *PA0109* |  | -1.797445199 |  |  |
| *PA0166* |  | 1.552218555 | xanthine permease |  |
| *PA0365* |  | -1.816686753 |  |  |
| *PA0545* |  | -1.554354797 | Preprotein translocase subunit SecD |  |
| *PA0565* |  | 2.331850483 |  |  |
| *PA0642* |  | 1.940351839 |  |  |
| *PA0786* |  | -2.363128107 | multi drug transporter |  |
| *PA0801* |  | -1.828083219 | iron regulated membrane protein |  |
| *PA0811* |  | -1.560712269 | major facilitator super family protein |  |
| *PA0877* |  | -1.54365467 | putative DNA-binding transcriptional regulator |  |
| *PA0957* |  | 1.761149855 | Acyl-coenzyme A thioesterase |  |
| *PA1847* |  | 1.633012057 | Fe/S biogenesis protein NfuA |  |
| *PA2095* |  | -1.823481739 |  |  |
| *PA2098* |  | 2.371434776 | alpha/beta hydrolase |  |
| *PA2214* |  | 2.056504702 | MFS transporter |  |
| *PA2251* |  | 1.580193842 |  |  |
| *PA2475* |  | -1.76858756 | probable cytochrome P450 |  |
| *PA2546* |  | -3.048489827 | probable ring-cleaving dioxygenase |  |
| *PA2575* |  | 1.802652422 |  |  |
| *PA2667* |  | 1.989956988 |  |  |
| *PA2668* |  | 1.516673348 |  |  |
| *PA2716* |  | 1.596026822 | probable FMN oxidoreductase |  |
| *PA2803* |  | 2.846371103 | Beta-phosphoglucomutase |  |
| *PA3015* |  | 1.683051533 |  |  |
| *PA3374* |  | -1.513687603 | Alpha-D-ribose 1-methylphosphonate 5-triphosphate diphosphatase PhnM |  |
| *PA3498* |  | -2.488764157 | probable oxidoredctase |  |
| *PA3500* |  | 1.552021928 |  |  |
| *PA3535* |  | -1.955483916 | probable serine protease |  |
| *PA3601* |  | -2.159889559 | rpmE2 ribosomal protein |  |
| *PA3674* |  | 1.700181592 |  |  |
| *PA3767* |  | -1.700670568 | tRNA(Arg) A34 adenosine deaminase TadA |  |
| *PA3952* |  | 1.788556912 |  |  |
| *PA4066* |  | 1.761707079 |  |  |
| *PA4080* |  | -1.796991582 | DNA-binding response regulator |  |
| *PA4096* |  | 3.092973404 | MFS transporter |  |
| *PA4122* |  | 2.330621436 |  |  |
| *PA4134* |  | 1.763971056 |  |  |
| *PA4178* |  | -1.560212825 | Preprotein translocase subunit SecD |  |
| *PA4337* |  | -2.40953017 |  |  |
| *PA4355* |  | -1.526322551 | Predicted arabinose efflux permease |  |
| *PA4387* |  | 1.840718036 |  |  |
| *PA4578* |  | 1.565144348 |  |  |
| *PA4830* |  | -3.122205298 | Thioesterase |  |
| *PA5540* |  | -3.099066973 |  |  |
| *pscT* |  | -1.596929578 | translocation protein in type III secretion | Wicke et al. 2021 Brandão et al.2020 |
| *rplT* |  | 1.566157825 | 50S ribosomal protein subunit |  |
| *rpmC* |  | 2.086761259 | ribosomal protein |  |
| *rsmA* |  | 1.530059865 | carbon storage regulator |  |
| **Differentially expressed *P. aeruginosa* genes only during JG024+JG005 coinfection at 8 minutes** | | | | |
|  | **JG024+JG005** | **JG024** | **Gene function** | **Similar observation in other studies** |
| *cupB2* | 2.025810994 |  | Pilus assembly protein |  |
| *fahA* | 1.644677875 |  | Fumarylacetoacetase |  |
| *lipA* | 2.063553435 |  | lactonizing lipase precursor |  |
| *mexH* | 1.642686535 |  | probable Resistance-Nodulation-Cell Division (RND) efflux membrane fusion protein precursor |  |
| *moaB1* | -1.54111774 |  | molybdenum cofactor |  |
| *oprP* | 1.941629039 |  | Phosphate-specific outer membrane porin OprP precursor |  |
| *osmC* | 1.925577756 |  | osmotically inducible protein OsmC |  |
| *PA0049* | 1.878023498 |  |  |  |
| *PA0057* | 1.514475184 |  |  |  |
| *PA0150* | -2.206306229 |  | ferric-dicitrate binding protein / membrane sensor |  |
| *PA0192* | 1.856987438 |  | TonB dependent |  |
| *PA0446* | 1.634319813 |  |  |  |
| *PA0682* | 2.523741474 |  | type II secretion system protein G, HxcX atypical pseudopilin | Brandão et al.2020 |
| *PA0699* | 1.826713224 |  | PpiC-type peptidyl-prolyl cis-trans isomerase |  |
| *PA0709* | 2.579147931 |  | quinol monooxygenase |  |
| *PA0737* | 1.572841178 |  |  |  |
| *PA0800* | -1.702154789 |  |  |  |
| *PA0978* | 2.018455213 |  |  |  |
| *PA1027* | 1.747955293 |  | NAD+-dependent alpha-aminoadipic semialdehyde dehydrogenase |  |
| *PA1153* | -1.846796642 |  | Prophage antirepressor |  |
| *PA1264* | 1.949913912 |  | probable transcriptional regulator |  |
| *PA1647* | -1.540513966 |  | Sulfate transporter |  |
| *PA1922* | 2.025810994 |  | TonB-dependent receptor |  |
| *PA2017* | -2.08748125 |  |  |  |
| *PA2073* | 1.697008854 |  | purine-cytosine permease |  |
| *PA2151* | 2.141307703 |  |  |  |
| *PA2208* | 1.826761714 |  | tricarboxylate transporter family |  |
| *PA2261* | 2.026147926 |  | 2-ketogluconate kinase |  |
| *PA2339* | 1.902664039 |  | probable binding-protein-dependent maltose/mannitol transport protein |  |
| *PA2340* | -1.797794914 |  | binding-protein-dependent maltose/mannitol transport protein |  |
| *PA2365* | 1.751459368 |  | Predicted component of the type VI protein secretion system |  |
| *PA2409* | 1.69457084 |  | ABC transporter permease |  |
| *PA2439* | 1.73930394 |  |  |  |
| *PA2465* | 1.776120849 |  | iron regulated membrane protein |  |
| *PA2528* | 1.63556423 |  | RND transporter |  |
| *PA2750* | -2.341296482 |  | Staphylococcus nuclease homolog |  |
| *PA2882* | 1.776281133 |  | histidine kinase, two component sensors |  |
| *PA3091* | -1.503679997 |  | chromosome segregation protein |  |
| *PA3133* | 1.929267501 |  | transcriptional regulator |  |
| *PA3144* | -1.599609971 |  | transposase with Helix-turn-helix Hin domain |  |
| *PA3232* | 1.877418973 |  | DNA polymerase III subunit epsilon |  |
| *PA3233* | 1.536459821 |  |  |  |
| *PA3428* | 3.4083363 |  |  |  |
| *PA3431* | -2.186681317 |  |  |  |
| *PA3588* | -2.097774694 |  | probable outer membrane porin |  |
| *PA3681* | -1.793361828 |  |  |  |
| *PA3840* | -1.640020575 |  | SAM-dependent methyltransferase |  |
| *PA3846* | -2.383866204 |  |  |  |
| *PA3907* | -1.652725337 |  | TOX-REase-5 domain-containing effector, TseT |  |
| *PA3953* | 1.924613967 |  |  |  |
| *PA4064* | 1.969025272 |  | probable ATP-binding component of ABC transporter |  |
| *PA4087* | 3.4083363 |  |  |  |
| *PA4111* | 2.348280663 |  |  |  |
| *PA4218* | 3.408175596 |  | siderophore transporter |  |
| *PA4611* | -1.90978286 |  |  |  |
| *PA4786* | 1.64332493 |  | probable short-chain dehydrogenase |  |
| *PA4826* | -1.607317922 |  |  |  |
| *PA4909* | -1.938574962 |  | ABC transporter |  |
| *PA5024* | -1.739045421 |  | sulfite transporter |  |
| *PA5097* | 1.65847318 |  | probable amino acid permease |  |
| *PA5115* | -1.575528647 |  | methyl transferase domain |  |
| *PA5539* | 2.140617483 |  | RidA subfamily protein |  |
| *pelC* | 2.140970637 |  |  |  |
| *potB* | -1.624613671 |  | polyamine transport protein PotB |  |
| *rhlI* | -1.585675118 |  | autoinducer synthesis protein RhlI |  |
| *tonB1* | -1.645276663 |  |  |  |
| *PA1664* | -2.809129302 |  |  |  |
| *phnW* | -2.809270307 |  | 2-aminoethylphosphonate:pyruvate aminotransferase |  |
| *PA3464* | -2.846327074 |  | zinc dependent phospholipase |  |
| *PA2069* | -2.933287806 |  | probable carbamoyl transferase |  |
| *coIII* | -3.075087413 |  | cytochrome c oxidase, subunit III |  |
| *PA1938* | -6.755240945 |  |  |  |

**Unique subset of differentially expressed *P. aeruginosa* genes during JG024 or JG024+JG005 co-infection at 24 minutes (these expression levels were not statistically at 8 minutes).**

| **Differentially expressed *P. aeruginosa* genes during both JG024 single infection and JG024+JG005 co-infection at 24 minutes** | | | | |
| --- | --- | --- | --- | --- |
|  | **JG024+JG005** | **JG024** | **Gene function** | **Similar observation in other studies** |
| *ackA* | -1.502871964 | -1.588604893 | Acetate kinase |  |
| *arsR* | 2.165664717 | 2.105023073 | ArsR family transcriptional regulator |  |
| *ccoO2* | -2.057062443 | -1.765700273 | Cytochrome c oxidase, cbb3-type, |  |
| *ccoP2* | -1.977522773 | -1.566909563 | Cytochrome c oxidase, cbb3-type, CcoP |  |
| *ccoQ2* | -1.927292279 | -1.696327474 | Cytochrome c oxidase, cbb3-type, CcoQ |  |
| *dhcB* | 2.66097091 | 2.805323529 | dehydrocarnitine CoA transferase, subunit B |  |
| *gpuP* | 2.253312799 | 2.492712962 | 3-guanidinopropionate transport protein |  |
| *hcnA* | -1.687585164 | -2.05701281 |  |  |
| *hcnB* | -2.49298506 | -2.301223773 |  |  |
| *hcnC* | -2.254196224 | -2.328402075 |  |  |
| *nirS* | -1.640549873 | -1.610538286 | nitrite reductase precursor |  |
| *PA0014* | 3.277899131 | 2.304068715 |  |  |
| *PA0471* | -2.288214055 | -1.542209013 | FiuR |  |
| *PA0515* | -1.711650759 | -2.356651871 | nirD |  |
| *PA0864* | 1.636991662 | 1.925774875 | probable transcriptional regulator |  |
| *PA0984* | 2.826051678 | 1.816479658 | colicin immunity protein |  |
| *PA1282* | 2.355155199 | 1.622358275 | probable major facilitator superfamily (MFS) transporter |  |
| *PA1540* | 2.188620609 | 2.347655919 |  |  |
| *PA1541* | 2.277833353 | 2.000946863 |  |  |
| *PA1592* | 3.274928472 | 2.047621955 |  |  |
| *PA1882* | 2.950502241 | 2.077290617 | probable cation transporter |  |
| *PA1889* | 2.09852428 | 2.00344428 | probable major facilitator superfamily (MFS) transporter |  |
| *PA1908* | -1.975229813 | -2.237968181 | probable major facilitator superfamily (MFS) transporter |  |
| *PA2036* | 2.112132716 | 2.100452439 | hypothetical protein |  |
| *PA2126* | -1.552595631 | -1.833235335 | cupA gene regulator C, CgrC |  |
| *PA2404* | 1.664845809 | 1.827358487 | FpvH |  |
| *PA2441* | 2.719096058 | 2.491257828 |  |  |
| *PA2557* | 2.463996611 | 1.959074874 | probable AMP-binding enzyme |  |
| *PA2650* | 2.086778181 | 2.87938097 |  |  |
| *PA2698* | 3.038420826 | 3.437276088 | probable hydrolase |  |
| *PA2702* | 1.972675732 | 1.653769498 | Tse2 ADP-ribosyltransferase toxins |  |
| *PA2935* | 2.382007651 | 2.285087079 |  |  |
| *PA3136* | 2.966500402 | 2.371348701 | Multidrug resistance efflux pump |  |
| *PA3265* | 1.550803018 | 1.523994732 | probable transporter |  |
| *PA3268* | -2.001900885 | -1.511493573 | probable TonB-dependent receptor |  |
| *PA3445* | 2.400175927 | 2.309213786 | sulfonate binding receptor |  |
| *PA3446* | 2.373150152 | 1.866205914 | NAD(P)H-dependent FMN reductase |  |
| *PA3450* | 1.531398841 | 1.747583187 | 1-Cys peroxiredoxin LsfA |  |
| *PA3661* | 1.834429679 | 2.287221818 |  |  |
| *PA3718* | 1.839177669 | 1.880283809 | probable major facilitator superfamily (MFS) transporter |  |
| *PA3741* | 1.914171696 | 1.840602925 | Acyl-CoA hydrolase |  |
| *PA3779* | 1.585588926 | 2.293953309 | putative periplasmic substrate binding protein |  |
| *PA3869* | 1.589091003 | 1.641374835 |  |  |
| *PA3912* | -1.74536226 | -2.085429618 | putative protease |  |
| *PA4182* | 2.184950025 | 2.374782151 | FMN-binding regulatory protein PaiB |  |
| *PA4287* | 1.859546152 | 1.590033573 | Permease of the drug/metabolite transporter |  |
| *PA4358* | -2.101430465 | -2.14822305 | ferrous iron transporter B |  |
| *PA4514* | -1.842604344 | -1.907321457 | probable outer membrane receptor for iron transport |  |
| *PA4523* | -2.140284094 | -1.607368579 |  |  |
| *PA4629* | 2.140394662 | 1.847390146 | Putative Ca2+/H+ antiporter |  |
| *PA4635* | 3.898518487 | 2.52802953 | uncharacteised membrane protein |  |
| *PA4739* | 1.627503928 | 1.629515517 | Osmotically-inducible protein OsmY |  |
| *PA4785* | 2.12647098 | 1.959506716 | acetyl-CoA acetyltransferase |  |
| *PA5083* | -2.271127941 | -2.237668119 |  |  |
| *PA5212* | 2.725984112 | 1.71393237 |  |  |
| *PA5530* | 2.940818305 | 2.835844602 | MFS dicarboxylate transporter |  |
| *pelA* | 2.018897979 | 1.605892025 |  |  |
| *xylS* | 1.55534796 | 1.977166743 | transcriptional regulator XylS |  |
| **Differentially expressed *P. aeruginosa* genes only with JG024+JG005 co-infection at 24 minutes** | | | | |
|  | **JG005+JG024** | **JG024** | **Gene function** | **Similar observation in other studies** |
| *algU* | 1.822101607 |  | sigma factor AlgU | Lood et al. 2020 and Wicke et al.2021 |
| *arnF* | -1.773548621 |  | 4-amino-4-deoxy-L-arabinose-phosphoundecaprenol flippase |  |
| *arr* | 1.895644214 |  | aminoglycoside response regulator |  |
| *betT1* | 1.713103629 |  | choline transporter |  |
| *ccoN2* | -1.652912688 |  | cytochrome C oxidase, cbb3-type |  |
| *ccpR* | -1.639446102 |  | cytochrome C551 peroxidase |  |
| *chpD* | -1.536125624 |  | transcriptional regulator |  |
| *citA* | 1.578787741 |  | citrate transporter |  |
| *cupC1* | 1.655537404 |  | fimbrilal subunit |  |
| *eddB* | 2.253312799 |  | extracellular DNA degradation protein |  |
| *glcE* | 1.757853985 |  | glycolate oxidase subunit |  |
| *hcpA* | 2.659854213 |  | type VI secretion system effector A |  |
| *hcpB* | -6.354472065 |  | type VI secretion system effector B |  |
| *lecB* | 3.276887666 |  | fucose-binding lectin PA-IIL |  |
| *maiA* | 1.692302625 |  | maleylacetoacetate isomerase |  |
| *mdcD* | -2.862419594 |  | malonate decarboxylase beta subunit |  |
| *mexG* | 1.823502503 |  | efflux protein |  |
| *msuE* | 2.383157756 |  | NADH-dependent FMN reductase MsuE |  |
| *mucA* | 1.740611194 |  | anti-sigma factor MucA | Lood et al. 2020 and Wicke et al. 2021 |
| *mucB* | 1.808586744 |  | negative regulator for alginate biosynthesis MucB | Lood et al. 2020 and Wicke et al. 2021 |
| *napC* | -2.339032481 |  | cytochrome c-type protein NapC | Lood et al. 2020 |
| *narL* | -1.7705212 |  | two-component response regulator NarL |  |
| *oprG* | -1.768725759 |  | Outer membrane protein OprG precursor |  |
| *oruR* | 1.524585648 |  | transcriptional regulator OruR |  |
| *PA0029* | 2.810438241 |  | probable sulfate receptor |  |
| *PA0137* | 3.280023124 |  | probable permease of ABC transporter |  |
| *PA0144* | -1.679285308 |  |  |  |
| *PA0189* | 2.329433091 |  | Outer membrane protein OprD |  |
| *PA0206* | 1.947209219 |  | spermidine/putrescine ABC transporter |  |
| *PA0221* | -1.942903718 |  | aspartate aminotransferase family protein |  |
| *PA0234* | 1.688945089 |  |  |  |
| *PA0284* | 1.651758753 |  | sulfur starvation response protein OscA |  |
| *PA0321* | -1.512363885 |  | Histone deacetylase class II a |  |
| *PA0327* | 2.190873007 |  |  |  |
| *PA0450* | 2.071129789 |  | phosphate transporter |  |
| *PA0460* | 1.717159265 |  |  |  |
| *PA0462* | 1.719321801 |  |  |  |
| *PA0489* | 2.562458392 |  | Predicted amidophosphoribosyltransferases |  |
| *PA0490* | 1.815292716 |  |  |  |
| *PA0499* | 1.650684621 |  | P pilus assembly protein, chaperone PapD |  |
| *PA0506* | 1.804212857 |  | acyl-CoA dehydrogenase |  |
| *PA0550* | 1.691589595 |  | Hydroxypyruvate isomerase |  |
| *PA0589* | 2.1481423 |  | thiosulfate sulfurtransferase |  |
| *PA0702* | 1.685967084 |  | Sterol desaturase/sphingolipid hydroxylase |  |
| *PA0712* | 1.735715923 |  | putative toxin-anti toxin pair |  |
| *PA0729* | 1.57026458 |  | ParE toxin of type II toxin-antitoxin system, |  |
| *PA0730* | 1.505447847 |  | Pimeloyl-ACP methyl ester carboxylesterase |  |
| *PA0797* | 1.626829699 |  | transcriptional regulator |  |
| *PA0813* | 3.126358248 |  | Selenocysteine lyase/Cysteine desulfurase |  |
| *PA0825* | 2.050354161 |  |  |  |
| *PA0833* | 1.590521015 |  | Outer membrane protein ompA |  |
| *PA0874* | 1.823298481 |  |  |  |
| *PA0909* | 2.005143401 |  | Putative 3TM holin, Phage_holin_3 |  |
| *PA0941* | 1.74710131 |  | Disulfide Oxidoreductases |  |
| *PA0977* | 3.729682809 |  |  |  |
| *PA1026* | 1.728830119 |  | transcriptional elongation factor |  |
| *PA1135* | -2.012616878 |  | protein deglycase HchA |  |
| *PA1137* | 2.530696509 |  | putative NAD(P)H quinone oxidoreductase, |  |
| *PA1215* | -2.261768055 |  |  |  |
| *PA1260* | 1.929035194 |  | ABC transporter periplasmic-binding protein, LhpP |  |
| *PA1394* | 2.600478302 |  |  |  |
| *PA1507* | 1.523540088 |  | xanthine permease |  |
| *PA1518* | 1.534335275 |  | 5-hydroxyisourate hydrolase |  |
| *PA1538* | 2.126041442 |  | flavin-containing monooxygenase |  |
| *PA1600* | 1.628846122 |  | probable cytochrome C |  |
| *PA1734* | 1.776698378 |  | cytochrome C |  |
| *PA1835* | 2.645018573 |  |  |  |
| *PA1874* | 2.731718338 |  |  |  |
| *PA1879* | 1.547902696 |  |  |  |
| *PA1892* | 2.543822972 |  |  |  |
| *PA2050* | 2.001913095 |  | RNA polymerase sigma factor |  |
| *PA2086* | -2.863025394 |  | epoxide hydrolase |  |
| *PA2145* | 2.3472992 |  |  |  |
| *PA2163* | -2.377737835 |  | 4-alpha-glucanotransferase |  |
| *PA2164* | 1.759058957 |  | **probable glycosyl hydrolase** |  |
| *PA2202* | 1.719792775 |  | probable amino acid permease |  |
| *PA2284* | 2.352335672 |  |  |  |
| *PA2294* | -3.030472388 |  | ABC transporter |  |
| *PA2297* | -3.030247824 |  | probable ferredoxin |  |
| *PA2310* | 3.059470946 |  | taurine oxygenase |  |
| *PA2314* | 3.160073561 |  | MFS transporter |  |
| *PA2348* | -2.261924037 |  | FMN-dependent oxidoreductase |  |
| *PA2364* | -1.52358795 |  |  |  |
| *PA2414* | -2.805718983 |  | L-sorbosone dehydrogenase |  |
| *PA2418* | 2.087319557 |  |  |  |
| *PA2670* | -2.431559417 |  |  |  |
| *PA2775* | 1.583322343 |  |  |  |
| *PA2779* | 1.767437867 |  |  |  |
| *PA2782* | 3.038612106 |  |  |  |
| *PA2834* | 1.795135938 |  | transcriptional regulator |  |
| *PA3031* | 2.120984772 |  |  |  |
| *PA3132* | 1.556720842 |  | probable hydrolase |  |
| *PA3522* | 1.689567479 |  | MexQ like efflux pump |  |
| *PA3597* | 1.77178599 |  | aminoacid permease |  |
| *PA3711* | -2.056920808 |  | transcriptional regulator |  |
| *PA3819* | 2.165309486 |  |  |  |
| *PA3889* | 1.777491317 |  | ABC transporter |  |
| *PA3931* | 2.368446076 |  | putative methionine-binding protein |  |
| *PA3938* | 2.230782692 |  | probable periplasmic taurine-binding protein precursor |  |
| *PA3962* | 1.863861653 |  |  |  |
| *PA4023* | 1.988706227 |  | ethanolamine permease |  |
| *PA4075* | 1.60535653 |  | SAM dependent methyl transferase |  |
| *PA4108* | -1.704210885 |  | cyclic di-GMP phosphodiesterase |  |
| *PA4150* | -2.261577931 |  | probable dehydrogenase E1 component |  |
| *PA4181* | 1.962291165 |  |  |  |
| *PA4437* | 3.158239352 |  |  |  |
| *PA4509* | -1.635392549 |  | Allophanate hydrolase subunit 2 |  |
| *PA4637* | 1.685581058 |  |  |  |
| *PA4692* | 1.596789536 |  |  |  |
| *PA4806* | 1.522716643 |  | transcriptional regulator |  |
| *PA4913* | 1.599060529 |  | ABC transporter |  |
| *PA4979* | 1.741085298 |  | acyl-CoA dehydrogenase |  |
| *PA5027* | -1.572441701 |  | universal stress family protein |  |
| *PA5168* | 1.693648045 |  | dicarboxylate transporter |  |
| *PA5220* | -3.152048443 |  |  |  |
| *PA5275* | 1.997278974 |  |  |  |
| *PA5462* | 1.616983612 |  |  |  |
| *PA5473* | 1.828854398 |  |  |  |
| *PA5475* | -1.662692461 |  |  |  |
| *PA5482* | 2.02659399 |  |  |  |
| *pcaK* | 2.087096506 |  | 4-hydroxybenzoate transporter PcaK |  |
| *pelD* | 1.805814756 |  |  |  |
| *plcN* | 1.609221185 |  | non-hemolytic phospholipase C precursor |  |
| *plcR* | 2.270815371 |  | phospholipase accessory protein PlcR precursor |  |
| *ppgL* | 2.053323618 |  | periplasmic gluconolactonase, |  |
| *pscG* | -1.505426897 |  | type III export protein |  |
| *rfaD* | -1.534754304 |  | ADP-L-glycero-D-mannoheptose 6-epimerase |  |
| *rpmF* | 1.601191587 |  | 50S ribosomal protein L32 |  |
| *rsaL* | -1.634564472 |  | regulatory protein RsaL |  |
| *wzz* | 1.77733743 |  | O-antigen chain length regulator |  |
| *xcpZ* | -1.887918272 |  | general secretion pathway protein M |  |
|  | | | | |
| **Differentially expressed *P. aeruginosa* genes only with JG024 infection at 24 minutes** | | | | |
|  | **JG005+JG024** | **JG024** | **Gene function** | **Similar observation in other studies** |
| *amiA* |  | 1.91277626 | N-acetylmuramoyl-L-alanine amidase |  |
| *arcB* |  | -1.514382492 | ornithine carbamoyltransferase, catabolic |  |
| *arcC* |  | -1.533473512 | carbamate kinase |  |
| *atuF* |  | -1.961102167 | geranyl-CoA carboxylase, alpha-subunit (biotin-containing) |  |
| *cdhC* |  | -3.092309745 | Carnitine dehydrogenase-related gene C |  |
| *hcpC* |  | 3.606894163 | secreted protein Hcp |  |
| *hpcC* |  | -1.95389135 | 5-carboxy-2-hydroxymuconate semialdehyde dehydrogenase |  |
| *kdpA* |  | 1.604714739 | potassium-transporting ATPase, A chain |  |
| *kdpB* |  | 1.552549462 | potassium-transporting ATPase, B chain |  |
| *kdpF* |  | 2.312629182 | potassium-transporting ATPase subunit |  |
| *nirJ* |  | -1.750028165 | heme d1 biosynthesis protein |  |
| *nrdB* |  | 1.684895888 | ribonucleotide reductase |  |
| *opmD* |  | 1.761237073 | probable outer membrane protein precursor |  |
| *oprB* |  | -6.686808878 | outer membrane porin precursor |  |
| *PA0111* |  | -2.834791468 |  |  |
| *PA0240* |  | 1.605227982 | probable porin |  |
| *PA0445* |  | -2.274698657 | probable transposase |  |
| *PA0452* |  | 1.707152704 | probable stomatin-like protein |  |
| *PA0510* |  | -1.678673467 | NirE, Uroporphyrin-III C-methyltransferase |  |
| *PA0512* |  | -1.650345455 | NirH |  |
| *PA0513* |  | -2.445458882 | NirG |  |
| *PA0734* |  | 1.711734879 |  |  |
| *PA0848* |  | 2.309292883 |  |  |
| *PA1051* |  | -1.824433908 | H+/gluconate importer |  |
| *PA1111* |  | 2.625008381 |  |  |
| *PA1154* |  | 6.391446517 |  |  |
| *PA1322* |  | -2.066066326 | probable TonB-dependent receptor |  |
| *PA1332* |  | 2.101537627 |  |  |
| *PA1410* |  | -2.778478593 | probable periplasmic spermidine/putrescine-binding protein |  |
| *PA1435* |  | -2.326148672 | RNA efflux protein |  |
| *PA1470* |  | 2.009679732 | probable short-chain dehydrogenase |  |
| *PA1500* |  | 3.498041808 | probable oxidoreductase |  |
| *PA1508* |  | -2.265415641 |  |  |
| *PA1566* |  | -1.960875579 | Glutamylpolyamine synthetase |  |
| *PA1873* |  | 1.513703535 |  |  |
| *PA1931* |  | -2.013495805 | Aerobic-type carbon monoxide dehydrogenase |  |
| *PA1977* |  | 1.880435058 | Permease of the drug/metabolite transporter |  |
| *PA2037* |  | 1.602951454 |  |  |
| *PA2055* |  | 2.369111906 | major facilitator superfamily (MFS) transporter |  |
| *PA2089* |  | -1.640913879 |  |  |
| *PA2138* |  | -1.816790153 | ATP dependent DNA ligase, LgD |  |
| *PA2307* |  | 1.790326238 | probable permease of ABC transporter |  |
| *PA2406* |  | 1.702546924 | Fpvk membrane protein, Iron acquiistion | Iron acquisition in *Pseudomonas aeruginosa* by the siderophore pyoverdine (Bonneau et al. 2020) |
| *PA2407* |  | 1.901331943 | FpvC membrane protein |  |
| *PA2408* |  | 1.534770294 | FpvD membrane protein |  |
| *PA2477* |  | -1.617743579 | thiol:disulfide interchange protein |  |
| *PA2690* |  | -2.25133003 | probable transposase |  |
| *PA2754* |  | -1.787892439 |  |  |
| *PA2786* |  | 3.225327822 |  |  |
| *PA2868* |  | -1.734137976 |  |  |
| *PA2918* |  | 2.39114595 | probable short-chain dehydrogenase |  |
| *PA3140* |  | 2.101156847 |  |  |
| *PA3338* |  | 2.267740678 |  |  |
| *PA3593* |  | 1.702244194 | probable acyl-CoA dehydrogenase |  |
| *PA3843* |  | 1.626184891 |  |  |
| *PA3871* |  | -1.920100569 | probable peptidyl-prolyl cis-trans isomerase, PpiC-type |  |
| *PA3936* |  | 1.807731053 | probable permease of ABC taurine transporter |  |
| *PA4022* |  | 1.674793529 | hydrazone dehydrogenase, HdhA |  |
| *PA4038* |  | 2.737598786 |  |  |
| *PA4139* |  | 3.117357703 |  |  |
| *PA4357* |  | -2.237174403 |  |  |
| *PA4714* |  | 1.587293067 |  |  |
| *PA4789* |  | 1.599124605 |  |  |
| *PA4805* |  | -1.531813731 | probable class III aminotransferase |  |
| *PA4828* |  | -2.937299878 |  |  |
| *PA4862* |  | -3.092878674 | probable ATP-binding component of ABC transporter |  |
| *PA4895* |  | -2.96914342 | probable transmembrane sensor |  |
| *PA4912* |  | 2.368921334 | branched chain amino acid ABC transporter membrane protein |  |
| *PA4990* |  | 3.372092314 | SMR multidrug efflux transporter |  |
| *PA5189* |  | -1.733529666 | probable transcriptional regulator |  |
| *PA5207* |  | -1.593868406 | probable phosphate transporter |  |
| *PA5231* |  | -1.517835918 | ATP-binding/permease fusion ABC transporter |  |
| *PA5325* |  | 1.828705476 | SphA. Deletion of sphA or sphR renders *P. aeruginosa* susceptible to killing by sphingosine |  |
| *pchD* |  | 1.702659422 | pyochelin biosynthesis protein |  |
| *pta* |  | -1.669608309 | phosphate acetyltransferase |  |
| *pvdE* |  | -1.52156193 | pyoverdine biosynthesis protein | Brandão et al. 2020 |
| *rmf* |  | -1.830455701 | ribosome modulation factor |  |
| *xqhA* |  | -1.54031463 | secretion protein XqhA |  |
