# Supplementary material for: Phage–phage competition and biofilms affect interactions between two virulent bacteriophages and Pseudomonas aeruginosa
Source: ISME J. 2025 Apr 6;19(1):wraf065. doi: 10.1093/ismejo/wraf065 (PMC12041424; doi:10.1093/ismejo/wraf065)
Supplement: Supplementary_Methods_wraf065 [file supplementary_methods_wraf065.docx]

**Phage-phage competition and biofilms affect interactions between two virulent bacteriophages and *Pseudomonas aeruginosa***

Magdalena Bürkle, Imke H. E. Korf, Anne Lippegaus, Sebastian Krautwurst, Christine Rohde, Chantal Weissfuss, Geraldine Nouailles, Xavière Menatong Tene, Baptiste Gaborieau, Jean-Marc Ghigo, Jean-Damien Ricard, Andreas C. Hocke, Kai Papenfort, Laurent Debarbieux, Martin Witzenrath, Sandra-Maria Wienhold, Gopinath Krishnamoorthy

**Supplementary Methods:**

**Double-agar overlay plaque assay (for phage enumeration and propagation):**

The term “indicator strain” refers to the specific P. aeruginosa strain used for individual phage titre determination. Indicator strains were selected according to their susceptibility to only one phage from a phage combination (as tested in this study) and comparable plating efficiency.

Optical density of bacterial growth was measured at 600 nm (OD_600_) or viable counts were enumerated from collected samples at specified time points. A double-agar overlay was performed for each phage with 100 µL of mid-log *P. aeruginosa* growth suspension (OD_600_ ≈ 0.2) of corresponding indicator strains (JG005, JG004 in *P. aeruginosa* F2230, JG024 and PTLAW1 in *P. aeruginosa* PA14; these and other strains used in this study are listed in **Supplementary Table S1**) in pre-warmed 4 mL of Soft Agar (0.7% w/v technical agar [BD], 5 mM Magnesium Sulfate (MgSO_4_) [Merck]) poured onto bottom Luria-Bertani (LB, Carl Roth, 6669.2) agar (1.5% w/v technical agar, [BD]) plates as previously described [1]. We did not see any prophage induction in any of the tested *P. aeruginosa* strains.

**Phage enumeration:** Samples and suspensions of interest were 10-fold serially diluted in Phage Buffer (100 mM Sodium Chloride [NaCl], 8 mM MgSO_4_, 50 mM Tris-Hydrochloride Tris-HCl, pH 7.5) and were spotted (4 µL/spot) onto the soft agar after solidification. Plates were incubated overnight at 37 °C and 5% CO_2_. Plaques on specific *P. aeruginosa* indicator strains were counted the next day and phage titres were calculated as follows:

*n* = number of plaques, *d* = dilution factor, PFU = Plaque forming Unit per mL:

$$Phage Enumeration= n*250*d=\frac{PFU}{mL}$$

**Phages propagation:** A modified protocol was used to prepare the phage stocks [2]. Briefly, for double-agar assays, 100 µL of phage dilutions were adjusted to 1E+05 PFU/mL before addition to soft agar suspended with the appropriate indicator strain (OD_600_ ≈ 0.2) **(Supplementary Table S1)**. After mixing, the suspension was transferred into petri plates containing LB agar and were incubated overnight at 37 °C, and 5% CO_2_. The next day, 2.5 mL of Phage Buffer was added to each plate, and the plates were sealed with parafilm and placed at 4 °C under orbital shaking conditions (50 rpm). After 8 hours of incubation, the liquid overlay containing phages and lysed bacterial remnants was collected. Chloroform was added 1:1000 volume/volume and incubated at room temperature for 15 min to release trapped viruses. The suspension was centrifuged at 3,345 x g for 15 min without break. The supernatant was then syringe filtered first with 0.8 and subsequently with 0.4, and 0.22 µm (ROTILABO^®^). Prior to storage at 4 °C, the phage titre of the filtrate was determined.

***P. aeruginosa*-phages interaction**

The following phage-to-bacteria ratios were used for each experimental condition.

| single administration | | | JG024+JG005 co-administration | | |  |  |
| --- | --- | --- | --- | --- | --- | --- | --- |
| **Experimental condition** | **Corresponding figures** | **JG024 or JG005: bacteria ratio** | **Phage (PFU/mL)** | Bacteria (CFU/mL) | JG024:JG005: bacteria ratio | Each phage (PFU/mL) | Bacteria (CFU/mL) |
| Planktonic culture | Figures 1A, 2A, 6A  Suppl. Figures S1B-C | 1:1 | 1E+07 | 1E+07 | 1:1:1 | 1E+07 | 1E+07 |
| Planktonic growth sequential phage treatment | Figure 6A | 1:1 | 1E+07 | 1E+07 | 1:1:1 | 1E+07 | 1E+07 |
| Microplate lysis curve | Suppl. Figure S6B | 1:1 | 1E+08 | 1E+08 | 1:1:1 | 1E+08 | 1E+08 |
| Infected A549 cell line | Figures 1B, 2B | Not applicable | | | 10:10:1 (high) | 1E+07 | 1E+06 |
|  |  | Not applicable | | | 1:1:10 (low) | 1E+05 | 1E+06 |
| Adsorption kinetics | Figure 3A | 1:50 | 1E+06 | 5E+07 | 1:1:50 | 1E+06 | 5E+07 |
| One-step growth curve | Figure 3B,  Suppl. Figure S2B | 1:50 | 1E+06 | 5E+07 | 1:1:50 | 1E+06 | 5E+07 |
| Dual-transcriptomics | Figures 3C-D | 5:1 | 2.5E+09 | 5E+08 | 5:5:1 | 2.5E+09 | 5E+08 |
| *P. aeruginosa* PAO1  Biofilm | Figures 4A, 5A | Not applicable | | | 10:10:10:1 (high) including Bhz17 | 1E+09 | 1E+08 |
|  |  | Not applicable | | | 1:1:1:10 (low) including Bhz17 | 1E+07 | 1E+08 |
| *P. aeruginosa* PAO1  Δ*retS* biofilm | Figures 4B, 5B | Not applicable | | | 10:10:10:1 (high) including Bhz17 | 1E+10 | 1E+09 |
|  |  | Not applicable | | | 1:1:1:10 (low) including Bhz17 | 1E+08 | 1E+09 |
| *P. aeruginosa* PAO1  Biofilm: sequential and meropenem | Figures 6B-C | Not applicable | | | 5:5:1 | 5E+08 | 1E+08 |
| Bacteriophage-insensitive mutants | Suppl. Figure S4A | 10:1 | 1E+09 | 1E+08 | Not applicable | | |

**Planktonic growth:** Indicated *P. aeruginosa* strains were routinely cultured overnight on BD^TM^ Columbia Agar with 5 % sheep blood and the following day inoculated into Luria-Bertani (LB, Carl Roth, 6669.2) broth and incubated at 37°C with orbital shaking (200 rpm). Bacterial growth was measured at 600 nm using a spectrophotometer (WPA, CO 8000). Prior to enumerating the phages, the samples were centrifuged with 0.22 µM Spin-X tubes (Sigma-Aldrich CLS8160, 6,200 x g, 1 min) to separate the phages from the bacteria.

**Microtitre plate-based lytic growth curve determination:** *P. aeruginosa* PAO1 was routinely cultured as mentioned above. Microtitre plate covers were treated with 2 to 3 ml of 0.05% Triton X-100 in 20% ethanol solution [3] to avoid condensation of water droplets on the inner surface of the plate. All phage combinations JG005+JG024 and JG005+JG024+Bhz17, and single phage Bhz17 were diluted to a titre of 2E+08 PFU/mL in LB media (2 mL), and PAO1 culture was diluted to a final count of 2E+08 CFU/mL. Phage dilutions and bacterial suspension were mixed 1:1 in a well plate and absorbance was read every 10 min at 600 nm (Molecular Devices SpectraMax M2e Multi-Mode Microplate Reader unit using regular settings) at 37 °C.

**A549 cell line infection:** A549 human type II alveolar epithelial cells (American Type Culture Collection; CCL-185^TM^) that had undergone 8-15 passages were used. Cells were cultured in antibiotic-free Dulbecco's Modified Eagle Medium containing 10% heat-inactivated foetal calf serum and 1% L-glutamine at 37 °C with 5% CO_2_. Cells were seeded the day before the infection into 12-well plates with a final cell density of 1E+05 cells/well. Chromatographically purified phage preparations (Fraunhofer Institute for Toxicology and Experimental Medicine, Germany) at indicated phage-to-bacteria ratio were used for A549 infection experiments only.

**Determination of the frequency of bacteriophage-insensitive mutants:** Bacteriophage-Insensitive Mutants (BIMs) frequencies were determined, as reported earlier [4]. Briefly, an equal volume of diluted log-culture (OD_600_ ≈ 0.2 or counted as 2E+08 CFU/mL) of the indicated *P. aeruginosa* and the phage of interest (2E+09 PFU/mL of each phage JG005, JG024, or combination of both) was poured into a soft-agar overlay. Number of surviving clones were counted after 24 h of incubation of plates at 37 °C and 5% CO_2_. The BIMs frequency was calculated as follows:

$BIMs frequency=\frac{number of surviving colonies}{original bacterial titer}$.

**Superinfection exclusion:** PAO1 was infected with either JG005 or JG024 at a high phage-to-bacteria ratio of 10:1. After 8 min of infection, samples were placed on ice for 1 min and subsequently washed two times with an ice-cold LB medium via centrifugation for 4 min at 16,200 x g. Subsequently, the pellet was dissolved with pre-warmed LB medium, and the cells were infected with the respective other phage at a phage-to-bacteria ratio of 1:10. Phage production was monitored for two hours every 24 min.

**Bacteria-phage dual RNA sequencing**

Transcriptomic analysis via bulk RNA sequencing was carried out using logarithmic growth of PAO1, which was either infected with phage JG024 alone and JG024 and JG005 combination at phage-to-bacteria ratio 5:1 to increase the chances of these two phages infecting co-infection of the same single cell while avoiding rapid bacteriolysis. After the addition of JG024 or JG024+JG005 at the indicated time point, the progress of phage infection was immediately stopped with stop solution (1%, 9% ethanol, 90% sample), pelleted and resuspended in TRIzol to lyse the cells, inactivate RNAs and initiate organic extraction. Additionally, the viability of both *P. aeruginosa* and each phage was enumerated prior adding stop solution.

**cDNA libraries preparation and sequencing:** Total RNA was isolated by hot-phenol method and digested with TURBO^TM^ DNase (Thermo Fisher Scientific). Ribosomal RNA was depleted using rRNA-specific biotinylated probes [5]. cDNA libraries were prepared using the NEBNext® Small RNA Library Prep Set for Illumina (NEB; #E7300L). cDNA libraries were sequenced using an Illumina NextSeq1000 system in single-read mode for 100 cycles. Demultiplexed raw reads were trimmed for quality, and 3’ adaptors using the CLC Genomics Workbench (Qiagen). Reads were mapped to PAO1, JG024, and JG005 genomic sequences (NCBI accession numbers: AE004091, NC_017674, PP712940), and unique gene reads mapped to the different organisms were compared to the total number of reads. Fold enrichment in the infected samples was compared to the untreated control using the CLC “Differential Expression for RNA-Seq” tool. Genes with a log_2_ fold change ≥ 1.5 and an FDR-adjusted *P* value ≤ 0.05 were defined as differentially expressed.

***P. aeruginosa* biofilm-phages interaction**

**Biofilm experiments:** Overnight grown culture of PAO1 or its Δ*retS* derivative was diluted (OD_600_ ≈ 0.1) and 200 µL was added into a round bottom microtitre plate (Sarstedt, micro test plate 96 well) and incubated at 37°C and 5% CO_2_ for 24 h in a humid chamber. Subsequently, the liquid phase was carefully pipetted off and the surface-attached biofilm was carefully washed three times with sterile phosphate-buffered saline (PBS, Gibco™) and then treated with phage combinations, 5 µg/mL of meropenem (Sigma-Aldrich) alone or in combination, or Phage Buffer.

The bacterial and phage counts were enumerated for the non-adherent and the adherent biofilm phases. First, the non-adherent population was carefully separated by pipetting and stored on ice for further processing. Second, the remaining adherent phase was washed three times with PBS (200 µL), followed by adding 100 µL of LB, and the mechanical dislodgement of the biofilm from the surface by vigorous scratching 30 times and pipetting up and down five times. The dislodgement step was repeated with another 100 µL LB, and the suspension was collected. Samples were serially diluted 10-fold, plated on blood agar plates for bacterial counts, and spotted on a double agar overlay with each indicator strain for individual phage counts.

**Microtitre biofilm assay: Crystal violet staining**

For biomass determination, the adherent phase was air-dried for 10 min after washing. After Crystal violet staining, the content solubilized in 98% ethanol and absorbance was measured at 594 nm (Molecular Devices SpectraMax M2e).

**Supplementary figures/table legends**

**Figure S1. Phages and their *P. aeruginosa* strain** **specificity. (A)** Strain-specific variation in *P. aeruginosa* phage susceptibility. Phage lysates (1E+06 PFU/mL) were 10-fold serially diluted, and 4 μL of each was spotted onto agar overlays prepared with the indicated *P. aeruginosa* strains. At least 70 plaques for each *P. aeruginosa* strain were selected for measuring the individual size using the plaque size tool [6]. Data are represented as violin blots. *P. aeruginosa* PAO1 Δ*retS* **(B)** and CHA **(C)** planktonic cultures were treated with JG005, JG024, JG005+JG024 combination (phage-to-bacteria ratio 1:1) or with Phage Buffer as control. Bacterial growth was monitored by measuring optical density at 600 nm (OD_600_), and corresponding phage titre were assessed by using specific *P. aeruginosa* indicator strains (**listed in** **Supplementary Table S1)**.

**Figure S2. Replication and growth parameters of each phage in different *P. aeruginosa* strains during single or co-administration.** **(A)** Phages JG004 and PTLAW1 are phylogenetically similar to JG005 and JG024, respectively. Both show comparable host range and plaque size on the indicated strains. Plaque size was calculated from 70 plaques/phage using the plaque size tool [6]. **(B)** When simultaneously added with JG004, the kinetics of JG024 and PTLAW1 are delayed. Data represent the mean ± SD of *n*=2 biological replicates.

**Figure S3. Superinfection exclusion.** Schematic illustration of the experimental setting to test if primary phage infection with one phage excludes superinfection with the second phage. A pre-established infection of PAO1 with JG005 or JG024 at a phage-bacterium-ratio of 10:1 for 8 min was superinfected with the respective other phage at a phage-bacterium-ratio of 1:10. Subsequently individual phage production was monitored every 24 min for two hours. Data represent the mean ± SD of *n*=3 biological replicates.

**Figure S4. Phage resistance frequency and phenotypic assessment. (A)** Frequency of bacteriophage-insensitive mutants (BIMs) from *P. aeruginosa* PAO1, PAO1 Δ*retS*, and CHA (phage-to-bacteria ratio 10:1). Statistical significance was assessed using ordinary two-way ANOVA and Šídák's multiple comparisons test to determine *P* values (not significant (*P* > 0.05) is not shown, **** *P* < 0.0001). **(B)** Representative JG005- and JG024-resistant PAO1 BIMs with altered phage susceptibility patterns **(see Supplementary Table S2)**. 10-fold dilutions of both phages were individually spotted on the lawn of the indicated BIMs, with JG005 (top) and JG024 (bottom). The phage concentration decreases from left to right, except for the JG024-resistant clones, where the phage concentration decreases from right to left. Therefore, these images were transposed to match the concentration gradient.

**Figure S5. Genomic comparison of JG005 and JG024. (A)** Genome organization of Pseudomonas phage JG005. The double-stranded DNA genome of phage JG005 is represented as a horizontal bar with vertical markers at every kilobase pair. Gene numbers are shown in boxes, which are shaded in black or grey according to their transcription from plus and negative strands, respectively. Putative gene functions and tRNA locations based on BLAST analyses are indicated. A Megablast analysis revealed that JG005 has close similarity to other phages (JG004, PAK_P1, PAK_P2, PAK_P4, PaP1, and vB_PaeM_C2-10_Ab1) infecting also *P. aeruginosa* PAO1*.* In particular, phages JG004 and JG005 are almost identical, and the difference between their DNA sequences is limited to a few nucleotides. **(B)** Genomic characteristics of JG005 and JG024. **(C)** Both JG005 and JG024 require *P. aeruginosa* transcriptional components for their replication. Rifampicin (Rif) is an inhibitor of RNA polymerase. Rif-treated PAO1 stalls the replication of both phages. Data represent the mean ± SD of *n*=2 biological replicates.

**Figure S6. Static *in vitro* biofilm production of *P. aeruginosa* PAO1 and PAO1 Δ*retS.* (A)** Schematic illustration of biofilm assay: Three *P. aeruginosa*-specific phages were each mixed at a 10-fold low/high phage-to-bacteria ratio, and this phage combination was tested on 24 h old *P. aeruginosa* PAO1 or the derivative Δ*retS* static biofilms in microtitre plates. Separated adherent (surface-attached) or non-adherent (dispersed) biofilm phases were used to determine bacterial and phage counts. The biomass of adherent biofilms was stained with crystal violet and measured at OD 590 nm. **(B)** Representative phage lysis curves of the non-replicative control phage Bhz17 individually (single) or in combination with JG005 and JG024 at a phage-bacteria (PAO1) ratio of 1:1. Bacterial growth (OD_600_) was continuously measured in plate reader for 24 h at 37°C. Data represent the mean ± SD of three biological replicates. **(C)** Crystal violet staining of biofilm biomass of the adherent compartment of PAO1 and PAO1 Δ*retS*. Data represent the mean ± SD of *n*=3 biological replicates from technical triplets. Statistical significance was assessed using Ordinary one-way ANOVA and Šídák's multiple comparisons test to determine *P* values (not significant (*P* > 0.05) is not shown, * *P* < 0.05).

**Supplementary Table S1**. Bacterial and phage isolates used in this study.

**Supplementary Table S2**. Gene polymorphisms in spontaneous JG005 and/or JG024 phage-resistant mutants of *P. aeruginosa* strains PAO1 and PA14.

**Supplementary Table S3**. Transcriptional responses of *P. aeruginosa* PAO1 and JG024 (with or without JG005 phage)

**Supplementary Methods**

**Sources**

1. Mazzocco A, Waddell TE, Lingohr E, Johnson RP Enumeration of bacteriophages by double agar overlay plaque assay. In: Clokie MR, Kropinski AM (eds.), *Bacteriophages. Methods and Protocols, Volume 1: Isolation, Characterization, and Interactions*, Humana Press, 2009, 69-76.

2. Pickard DJJ Preparation of bacteriophage lysates and pure DNA. In: Clokie MR, Kropinski AM (eds.), *Bacteriophages. Methods and Protocols, Volume 2: Molecular and Applied Aspects*, Humana Press, 2009, 3-9.

3. Brewster JD. A simple micro-growth assay for enumerating bacteria. *J Microbiol Methods*. 2003;**53**:77-86. <https://doi.org/10.1016/S0167-7012(02)00226-9>

4. O'flynn G, Ross R, Fitzgerald G, Coffey A. Evaluation of a cocktail of three bacteriophages for biocontrol of *Escherichia coli* O157: H7. *Appl Environ Microbiol* 2004;**70**:3417-24. <https://doi.org/10.1128/AEM.70.6.3417-3424.2004>

5. Culviner PH, Guegler CK, Laub MT. A simple, cost-effective, and robust method for rRNA depletion in RNA-sequencing studies. *mBio*. 2020;**11**:e00010-20. <https://doi.org/10.1128/mBio.00010-20>

6. Trofimova E, Jaschke PR. Plaque Size Tool: An automated plaque analysis tool for simplifying and standardising bacteriophage plaque morphology measurements. *Virology*. 2021;**561**:1-5. <https://doi.org/10.1016/j.virol.2021.05.011>
